# Screening the Extract of *Laportea bulbifera* (Sieb. et Zucc.) Wedd. Based on Active Component Content, Its Antioxidant Capacity and Exploration of Hepatoprotective Activity in Rats

**DOI:** 10.3390/molecules28176256

**Published:** 2023-08-25

**Authors:** Jiaxin Feng, Yue Sun, Zhongbao Wei, Hui Sun, Li Li, Junyi Zhu, Guangqing Xia, Hao Zang

**Affiliations:** 1 College of Pharmacy, Yanbian University, Yanji 133000, China; 13630304082@163.com (J.F.); 15981309048@163.com (Y.S.); lili1984@thnu.edu.cn (L.L.); qingguangx@thnu.edu.cn (G.X.); 2 Green Medicinal Chemistry Laboratory, School of Pharmacy and Medicine, Tonghua Normal University, Tonghua 134002, China; sunhui9405@163.com (H.S.); swx0527@163.com (J.Z.); 3 Institute of Scientific and Technical Information of Jilin, Changchun 130033, China; 13894531050@163.com; 4 Key Laboratory of Evaluation and Application of Changbai Mountain Biological Gerplasm Resources of Jilin Province, Tonghua 134002, China

**Keywords:** *Laportea bulbifera* (Sieb. et Zucc.) Wedd., phytochemical analysis, antioxidant activity, liver damage

## Abstract

*Laportea bulbifera* (Sieb. et Zucc.) Wedd., a plant with a long history of medicinal use, possesses uncertainly defined medicament portions while its antioxidant capacity remains largely unexplored. To gain a better understanding of its medicinal value, this study focused on investigating the *Laportea bulbifera* aboveground part (LBAP) and the *Laportea bulbifera* root (LBR). Through an assessment of the bioactive compound content, a significant finding emerged: the LBR exhibited notably higher levels of these bioactive phytochemicals compared to the LBAP. This observation was further reinforced by the antioxidant assays, which demonstrated the superiority of the LBR’s antioxidant capacity. The experimental results unequivocally indicate that the root is the optimal medicament portion for *Laportea bulbifera*. Furthermore, it was discovered that the presence of alcohol in the extraction solvent significantly enhanced the extraction of active ingredients, with the methanol extract of LBR performing the best among the extracts tested. Consequently, this extract was selected for further research. Leveraging cutting-edge UHPLC-ESI-Q-TOF-MS technology, the methanol extract of LBR was meticulously analyzed, revealing the presence of 41 compounds, primarily belonging to the phenolics and fatty acids. Remarkably, stability experiments demonstrated that the phenolics in the methanol extract maintained their stability across various pH values and during in vitro simulations of the human digestive system, albeit showing gradual degradation under high temperatures. Furthermore, the oxidative stability tests conducted on oils revealed the potential of the methanol extract as a stabilizer for olive oil and sunflower oil. Moreover, oral acute toxicity studies confirmed the low toxicity of the methanol extract, further supporting its safe use for medicinal purposes. Of particular note, histopathological examination and biochemical analysis affirmed the remarkable protective effects of the methanol extract against d-galactosamine-induced liver damage. These findings underscore the therapeutic potential of the methanol extract from the LBR in the treatment of diseases associated with oxidative imbalance.

## 1. Introduction

*Laportea Gaudich*., a genus of the Urticaceae family, comprises 28 plant species primarily found in subtropical and tropical regions such as China, Bangladesh, Cameroon, and Zimbabwe. Notably, this genus possesses toxic spines on both stems and leaves, which, upon contact, rapidly induce skin reactions in mammals, including erythema, itching, and tingling sensations [1]. Extensive research has led to the identification of various compounds within *Laportea Gaudich*., including flavonoids, coumarins, phenylpropanoids, and volatile oils. These chemical constituents have been thoroughly studied, unveiling numerous pharmacological properties such as hypoglycemic, antibacterial, anti-inflammatory, analgesic, anti-rheumatic, anti-ulcer, and antioxidant activities [2].

**Figure 1:**
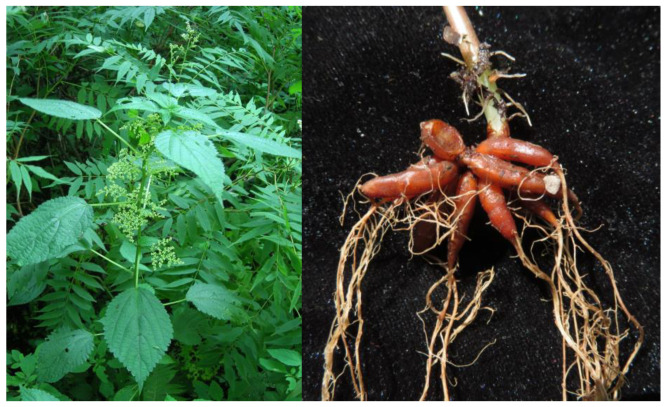
Morphology of *Laportea bulbifera* aboveground part and root.

Of particular significance within the *Laportea* genus is *Laportea bulbifera* (Sieb. et Zucc.) Wedd. (*L. bulbifera*), a diverse and economically important plant. *L. bulbifera* is known by various names including *Laportea elevata*, *Laportea terminalis*, and *Laportea sinensis*. It exhibits a wide distribution, primarily spanning regions of northeast and southwest China, as well as Japan, North Korea, Russia, and India. This adaptable plant thrives in diverse habitats, including forest understories, peripheries, and humid areas of semi-shady slopes, typically found at altitudes ranging from 1000 to 2400 m. *L. bulbifera* displays distinct phenological patterns, with its flowering period occurring between June and August, followed by the fruiting period from August to December, ensuring its presence throughout the year [3]. The medicinal importance of *L. bulbifera* is recognized notably among the Miao and Buyi ethnic minorities in Guizhou Province, China. The medicament portions of *L. bulbifera* include the whole plant or roots (Figure 1), which can be consumed orally or applied externally. It possesses several medicinal properties, including dispelling wind and eliminating dampness, promoting blood circulation, and removing stasis. *L. bulbifera* is commonly used to treat various conditions, including rheumatic arthralgia, limb numbness, fatigue-induced internal imbalances, nephritis dropsy, traumatic injuries, fractures, and irregular menstruation [4]. Extracts derived from *L. bulbifera* are frequently incorporated into pharmaceutical products, such as ointments and capsules. Notable examples include Runzao Antipruritic Capsules, Liuwei Shangfuning Ointments, and Fufang Shangning Ointments. Runzao Antipruritic Capsules, in particular, have gained significant popularity in the Chinese market, attesting to the effectiveness and desirability of *L. bulbifera*-based remedies [5].

The chemical constituents of *L. bulbifera* are highly complex, comprising an impressive collection of over 200 compounds, including flavonoids, coumarins, steroids, phenylpropanoids, fatty acids, and other bioactive substances [4,5]. This extensive range of compounds suggests that *L. bulbifera* may possess significant antioxidant properties. However, research on *L. bulbifera*’s antioxidant activity remains relatively limited, with only two published reports investigating its potential in scavenging 2,2-diphenyl-1-picryl hydrazyl (DPPH) radicals. These studies have explored various extracts, such as the aqueous, petroleum ether, and ethyl acetate extracts of the whole plant, as well as the aqueous and ethyl acetate extracts of the roots [5,6]. Consequently, the investigation of *L. bulbifera*’s antioxidant capacity is still in its early stages.

To address this knowledge gap, the present study aims to comprehensively evaluate the effectiveness of both the aboveground and underground parts of *L. bulbifera*. This assessment will encompass multiple criteria, including the content of active ingredients and the antioxidant capacity of each part. Additionally, advanced ultra-high performance liquid chromatography-mass spectrometry (UHPLC-MS) technology has been employed to identify and analyze the chemical constituents present in the extract. This sophisticated analytical approach will provide a deeper understanding of *L. bulbifera*’s medicinal properties, establishing a solid foundation for its potential clinical applications. In summary, through a comprehensive evaluation of *L. bulbifera*’s antioxidant properties and chemical composition, this study aims to uncover the potential health benefits offered by this remarkable plant.

## 2. Results and Discussion

This study conducted a comprehensive investigation of the *L. bulbifera* aboveground part (LBAP) and *L. bulbifera* root (LBR) using qualitative and quantitative phytochemical analysis, as well as in vitro antioxidant activity tests. Notably, the methanol extract of LBR exhibited the highest yield among all the extracts, indicating a rich concentration of multiple active ingredients. Moreover, it consistently demonstrated excellent performance in various antioxidant assays, further cementing its selection for in-depth research. The qualitative analysis utilizing UHPLC-MS identified a total of 41 compounds present in the methanol extract. Additionally, stability experiments conducted on the methanol extract revealed that the phenolics present exhibited notable stability across various pH levels and during in vitro simulations of the human digestive system. However, they did display gradual degradation when exposed to high temperatures. Furthermore, the oil stability experiments demonstrated the extract’s potential as a candidate for becoming an oil stabilizer. The evaluation of oral acute toxicity in mice demonstrated a low level of toxicity associated with the methanol extract, indicating its relative safety for medicinal use. Moreover, in an experimental model of d-galactosamine-induced liver damage in rats, the extract from LBR exhibited significant hepatoprotective effects, as confirmed through histopathological examination.

### 2.1. Qualitative Phytochemical Analysis

In this study, a comprehensive qualitative phytochemical analysis was conducted on LBAP and LBR. The results revealed that both LBAP and LBR contained proteins/amino acids, carbohydrates, phenolics, organic acids, tannins, alkaloids, and volatile oils and fats. However, neither LBAP nor LBR contained saponins, terpenoids, anthraquinones, cardiac glycosides, or cyanogenic glycosides. LBAP was found to contain triterpenoids, whereas LBR contained steroids. Furthermore, *LBR* does not contain flavonoids, but LBAP does contain flavonoids. On the other hand, LBR contained coumarins and lactones, which were absent in LBAP (Table 1).

### 2.2. Yields

To extract the bioactive compounds, both LBAP and LBR were subjected to extraction using four different solvents (water, methanol, ethanol, and 80% ethanol). The yield of LBR (ranging from 14.30 ± 1.29% to 24.90 ± 2.03%) was found to be higher than that of LBAP (ranging from 7.16 ± 0.93% to 16.60 ± 1.21%) (Table 2). This suggests that the LBR extract contains a relatively higher concentration of compounds compared to the LBAP extract. A study has shown that the yield of ethanol extract of LBR is 17.5%, which is lower than our results (21.86 ± 2.98%). This may be due to differences in solvent dosage and extraction time [7]. Among the solvents used, the aqueous extract of LBAP exhibited the highest yield, which can be attributed to its rich content of water-soluble components such as phenolics, proteins, and carbohydrates. However, it is vital to note that while the aqueous extract had the highest yield, it may also contain fewer effective ingredients like pigments and pectin, which could potentially impact compound identification and biological activity. On the other hand, the methanol extract of LBR showed the highest yield, likely due to the extraction of active ingredients such as phenolics, flavonoids, coumarins, and others.

### 2.3. Quantitative Phytochemical Analysis

#### 2.3.1. Total Carbohydrate Content (TCC)

Carbohydrates play a crucial role as essential biomolecules with diverse biological functions. Apart from serving as a source of energy, carbohydrates have been extensively studied for their various biological activities and therapeutic potential in treating different diseases [8]. Glycosides, in particular, have shown significant pharmacological effects [9]. Both LBAP and LBR were found to contain flavonoid glycosides, with LBR also containing phenolic glycosides [5,10]. In this study, we evaluated the carbohydrate content of different extracts from LBAP and LBR. As shown in Table 3, the TCC of LBAP ranged from 98.54 ± 0.51 to 172.38 ± 3.68 mg glucose equivalents (GE)/g extract. In contrast, the TCC of LBR ranged from 466.11 ± 4.43 to 686.14 ± 3.53 mg GE/g extract, indicating that LBR contains several times more carbohydrates than LBAP. This also means that regardless of whether it is LBAP or LBR, the TCC of the methanol extract was higher than that of the aqueous extract. This suggests that the detection method used in this experiment not only determines the content of carbohydrates but also allows estimation of the content of glycosides. Considering the solubility of solvents, it can be inferred that the TCC of the aqueous extracts mainly consists of carbohydrates, while the TCC of the methanol extracts mainly consists of glycosides.

#### 2.3.2. Total Protein Content (TP_ro_C)

Plant proteins offer numerous health benefits and serve as a valuable source of nutrition, being easily digested and absorbed by the human body. They exhibit various biological activities associated with health promotion, such as anti-inflammatory, antimicrobial, antidiabetic, and antioxidant properties [11]. In our study, the aqueous extract of LBAP showed a relatively high TP_ro_C of 440.23 ± 7.57 mg bovine serum albumin equivalents (BSAE)/g extract. Remarkably, the aqueous extract of LBR demonstrated an even higher TP_ro_C, reaching an astonishing 2032.86 ± 2.55 mg BSAE/g extract (Table 3). This significant protein content has the potential to not only enhance a patient’s physical health but also promote the absorption and regression of inflammatory edema in the hyperostosis area, thereby alleviating the symptoms associated with hyperostosis. These findings provide insights into why *L. bulbifera* has been traditionally used as a treatment for hyperostosis [4].

#### 2.3.3. Total Phenolic Content (TP_he_C)

Plant polyphenols possess abundant pharmacological activity and are believed to provide multiple benefits for human health. They exhibit a strong antioxidant capacity and have been shown to have beneficial effects on various health conditions including cancer, diabetes, and cardiovascular diseases [12]. In line with the findings on TCC and TP_ro_C, there exists a significant disparity in TP_he_C between LBAP and LBR. The TP_he_C of LBAP ranged from 23.69 ± 0.23 to 50.79 ± 0.90 mg gallic acid equivalents (GAE)/g extract, whereas the TP_he_C of LBR ranged from 98.62 ± 0.31 to 313.83 ± 4.16 mg GAE/g extract. The presence of tea polyphenols and danshen polyphenols is known to promote vasodilation, reduce inflammatory reactions, and prevent the formation of blood clots, all of which contribute to the prevention of cardiovascular disease [12,13]. This is consistent with the traditional efficacy of *L. bulbifera*, which emphasizes its ability to promote blood circulation and remove stasis and highlights the role of its rich polyphenolic content in these health benefits.

Furthermore, our experimental results suggest that solvents containing alcohol are more effective for extracting polyphenols from *L. bulbifera*. Extraction solvents such as methanol, ethanol, or 80% ethanol demonstrated a TP_he_C approximately three times higher than that of the aqueous extract. Traditionally, water has been the primary extraction solvent for *L. bulbifera* [5]. However, these findings indicate that incorporating a certain proportion of ethanol into the extraction process could yield higher amounts of polyphenols, potentially enhancing their efficacy.

#### 2.3.4. Total Flavonoid Content (TFC)

Flavonoids, which are natural polyphenolic substances found in vegetables, fruits, grains, and tea, are abundant in nature. These flavonoids play essential roles in various biological processes and are commonly included in human diets. They are known for their antimicrobial, antioxidant, and anti-inflammatory properties, which help reduce the risk of various diseases [14]. However, after analysis, we found that the various extracts of LBR did not contain flavonoids. On the other hand, moderate levels of TFC ranging from 4.17 ± 0.18 to 69.53 ± 1.25 mg quercetin equivalents (QE)/g extract were found in the different extracts of LBAP (Table 3). This indicates that different plant parts may have distinct chemical compositions, resulting in variations in medicinal properties. Consistent with existing literature reports, flavonoids are typically isolated from LBAP or the whole plant [10,15]. It is worth noting that only one study reported the isolation of flavonoids from the ethyl acetate layer of LBR [11]. This discrepancy may be attributed to the significant variation in the content of bioactive constituents across different habitats and the potential influence of harvesting times on the accumulation of these constituents [16].

#### 2.3.5. Total Phenolic Acid Content (TPAC)

In addition to evaluating TP_he_C, this study also assessed the TPAC of different extracts of LBAP and LBR. As shown in Table 3, LBR exhibited slightly higher TPAC values compared to LBAP. The TPAC of LBAP ranged from 4.85 ± 0.33 to 12.22 ± 1.20 mg caffeic acid equivalents (CAE)/g extract, while the TPAC of LBR ranged from 8.46 ± 0.65 to 18.46 ± 2.16 mg CAE/g extract. Previous literature points out that phenolic acids primarily include hydroxybenzoic acid, hydroxycinnamic acid, chlorogenic acid, and their isomers [5,10,17].

#### 2.3.6. Total Tannin Content (TT_an_C), Gallotannin Content (GC) and Condensed Tannin Content (CTC)

Tannins, found widely in plants, serve as defense mechanisms against predation and may regulate plant growth. They can be classified into two major groups: hydrolyzable tannins and condensed tannins, with gallotannin being a representative of the former [18]. In order to explore the tannin content in various extracts of LBAP and LBR, we measured the GC, CTC, and TT_an_C (Table 3). Similar to the components discussed earlier, LBR exhibited tannin content several times higher than LBAP. The experimental results reveal that LBR is predominantly rich in condensed tannins, with concentrations ranging from 55.23 ± 0.74 to 188.70 ± 0.43 mg GAE/g extract. Similar to TP_he_C, the use of solvents containing alcohol proves advantageous for the extraction of tannins.

This study unveils the potential of *L. bulbifera* as a rich source of bioactive compounds, including carbohydrates, glycosides, plant proteins, phenolics, phenolic acids, and tannins, each of which plays distinct physiological and pharmacological roles. One remarkable finding that has captured our attention is the significantly higher content of these bioactive phytochemicals in LBR compared to LBAP. It is well-established that the concentration of active ingredients directly impacts their pharmacological efficacy [19]. Hence, the notable divergence in active ingredient content between LBR and LBAP implies a significant difference in their respective efficacies.

This study has made a significant contribution by shedding light on a long-debated topic regarding the medicament portions of *L. bulbifera*. Traditionally, the use of both the whole plants and roots of *L. bulbifera* as medicament portions has prevailed, although without a scientific basis. Through our thorough examination of the active ingredient content, we have unequivocally established that the medicinal efficacy of LBR surpasses that of the whole plant. This is due to the substantially higher concentration of active compounds present in LBR compared to LBAP. The weight proportion of LBR, when using the whole plant, is limited to around 20%, while LBAP comprises approximately 80%, which significantly impacts the overall efficacy. Therefore, based on the active ingredient content, we advocate for the correct utilization of *L. bulbifera* roots in medicine. The reasoning behind these findings is readily understandable. *L. bulbifera* is a perennial herb, experiencing annual cycles of aboveground growth, withering, and subsequent regrowth. As a consequence, the accumulation of active ingredients in the aboveground parts is not substantial. In contrast, the roots persist underground throughout the lifespan of the plant, facilitating the effective accumulation of active compounds over time, resulting in their relatively higher content. This remarkable discovery fills a crucial gap in the existing literature and presents an intriguing insight into *L. bulbifera’s* medicinal properties. These findings hold important implications for the practical usage of *L. bulbifera* in healthcare applications. By focusing on the roots as the primary medicinal component, practitioners can maximize the therapeutic benefits of *L. bulbifera*, ensuring the administration of a higher concentration of active ingredients. Moreover, this discovery prompts further investigations into the specific active ingredients enriched in LBR, their metabolism pathways, and their corresponding physiological effects. Additionally, exploring the optimal extraction methods to isolate and concentrate these potent bioactive ingredients would pave the way for formulating *L. bulbifera*-based medicinal products with enhanced efficacy and improved patient outcomes. This paradigm shift in understanding the medicament portions of *L. bulbifera* opens up promising avenues for research and application in the field of herbal medicine.

### 2.4. Antioxidant Activity In Vitro

#### 2.4.1. DPPH and 2,2’-Azino-bis(3-ethylbenzothiazoline-6-sulphonic acid) Diammonium Salt Cation Radicals (ABTS) Tests

Evaluating the free radical scavenging capacity of natural compounds and extracts is essential for understanding their antioxidant potential. In vitro assessments often employ fat-soluble DPPH and water-soluble ABTS, which are widely recognized as standard indicators for measuring free radical scavenging ability [20].

The experimental results show that different extracts of LBAP have good free radical scavenging ability. At the same time, different extracts of LBR have better scavenging ability. With the exception of the aqueous extract, the other three LBR extracts exhibited DPPH scavenging ability that surpassed that of butylated hydroxytoluene (BHT), even matching the performance of trolox (Table 4). This consistent trend was also evident in the ABTS experiment. Remarkably, the ABTS scavenging ability of the three non-aqueous LBR extracts not only outperformed BHT but even surpassed trolox. These compelling experimental results can be attributed to the abundance of polyphenols present in LBR.

Additionally, our findings align with previous studies on *Laportea* species. Specifically, the methanol extracts of *Laportea alatipes* and *Laportea meyeniana* demonstrated commendable DPPH scavenging ability, further reinforcing the potential antioxidant properties of the *Laportea* genus [21,22]. These results collectively highlight the substantial antioxidant potential offered by LBR and related *Laportea* species, making them promising subjects for further investigation and exploitation in natural product research.

#### 2.4.2. Hydroxyl Radicals and Superoxide Radicals Tests

Assessing the antioxidant capacity of a compound requires investigating hydroxyl radicals and superoxide radicals generated within the body. The ability to scavenge these radicals plays a significant role in the detoxification of antioxidants and their effectiveness in minimizing cellular oxidative toxicity [23].

According to our research findings, the ethanol extract of LBAP exhibited the highest hydroxyl radical scavenging activity. However, its potency was still inferior to the three alcoholic extracts of LBR, which demonstrated equal or even better scavenging ability than the reference compound, BHT (Table 4). In terms of superoxide radicals, the various extracts of both LBAP and LBR displayed limited scavenging activity (Table 4). Interestingly, prior studies have highlighted the capacity of *Laportea aestuans* to scavenge both hydroxyl and superoxide radicals, further supporting the importance of these properties in evaluating the antioxidant potential of compounds [24,25].

#### 2.4.3. Ferric-Reducing Antioxidant Power (FRAP) and Cupric Ion Reducing Antioxidant Capacity (CUPRAC) Tests

The ability of the sample to reduce iron and copper ions could also reflect its antioxidant capacity [26]. The FRAP and CUPRAC assays are two commonly employed methods for this purpose. Our experimental results in the FRAP assay showed a significant positive correlation with the DPPH experimental results, underscoring a significant disparity between LBAP and LBR. With the exception of the aqueous extracts, the alcohol extracts of LBR demonstrated approximately five times higher ferrous ion reduction ability compared to LBAP. This gap was even more pronounced in the CUPRAC assay, where the copper ion reduction ability of the LBR extracts reached levels 20–30 times that of LBAP. Notably, the methanol and ethanol extracts of LBR exhibited performance similar to trolox, a well-known antioxidant (Table 5). These findings reinforce the literature, which has identified *Laportea alatipes* as possessing commendable antioxidant properties based on FRAP assays [21].

#### 2.4.4. Metal Chelating Tests

The presence of iron and copper ions can enhance the Fenton reaction, leading to increased oxidative stress in vivo [27]. Consequently, the identification of natural and effective metal ion chelating agents is of paramount importance.

Regrettably, both LBR and LBAP displayed weak iron chelating ability. However, in terms of copper chelation, the different extracts of LBR demonstrated superior chelating capacity, while the different extracts of LBAP exhibited moderate chelating ability (Table 5). These results suggest that LBR possesses selective copper ion chelation properties, which may have implications for its potential applications as a metal ion chelating agent.

#### 2.4.5. Hydrogen Peroxide (H_2_O_2_) and Singlet Oxygen Tests

H_2_O_2_ is a powerful oxidizing agent that is produced as a byproduct of human metabolism. While it has important roles in cellular signaling and immune responses, excessive levels of H_2_O_2_ can lead to oxidative stress and cell damage. Therefore, the effective scavenging of H_2_O_2_ is crucial for maintaining physical health [28]. Similarly, singlet oxygen is another reactive oxygen species (ROS) that could cause cellular damage. It is generated during various physiological and pathological processes, including inflammation and exposure to ultraviolet radiation [29].

Our experimental findings revealed that all extracts of LBAP demonstrated weak scavenging activity against H_2_O_2_. However, among the three extracts of LBR, except for the aqueous extract, good scavenging activity was observed, comparable to that of the positive control, gallic acid (Table 6). Interestingly, our experiment did not show any scavenging activity for singlet oxygen in the various extracts of LBAP and LBR (Table 6). To our knowledge, there is no literature reporting about the scavenging activity of *Laportea* plants against singlet oxygen.

#### 2.4.6. Hypochlorous Acid (HClO) Tests

HClO, a potent oxidant, plays a critical role in protecting the body against pathogens and aiding in wound healing. However, excessive production of HClO has been implicated in various human diseases [30].

In our study, various extracts of LBAP exhibited weak or no scavenging ability against HClO. Conversely, the aqueous extract of LBR showed good scavenging ability, and the other three extracts of LBR performed even better, surpassing the positive control, lipic acid, with the 80% ethanol extract outperforming trolox (Table 6). Hence, it is plausible that the extracts of LBR may contain natural compounds that could help eliminate excessive HClO in the body.

#### 2.4.7. Nitric Oxide (NO) Tests

NO is a gaseous molecule that freely diffuses through biological membranes and participates in various physiological processes. However, the accelerated production of NO can trigger the activation of inducible nitric oxide synthase, leading to damage to the intestinal membrane [31].

**Figure 2:**
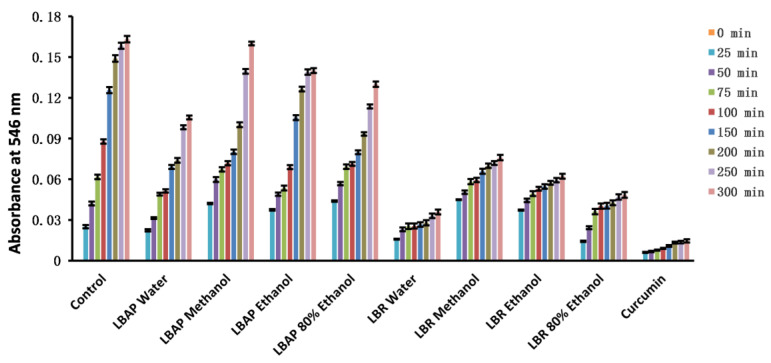
Changes in the absorbance of various extracts of *Laportea bulbifera* over time measured using the nitric oxide scavenging method. LBAP: *Laportea bulbifera* aboveground part. LBR: *Laportea bulbifera* root.

In our experiments, various extracts of LBR demonstrated good scavenging ability against NO (Figure 2). Among these extracts, the aqueous extract showed the best performance, closely resembling the positive control, curcumin. On the other hand, various extracts of LBAP exhibited weak scavenging ability against NO.

This study aimed to investigate the antioxidant potential of *L. bulbifera*, a plant known for its remarkable scavenging effects on various ROS. Our findings demonstrated the ability of *L. bulbifera* extracts to scavenge different types of ROS, including free radicals (DPPH, ABTS, hydroxyl, and superoxide radicals), reduce metal ions (FRAP and CUPRAC), chelate metal ions (iron and copper), and scavenge oxidants (H_2_O_2_, HClO, and NO). Specifically, we observed significantly stronger antioxidant capacity in the three extracts of LBR compared to LBAP, except for the aqueous extract. Notably, the methanol extract of LBR exhibited superior antioxidant capacity, often surpassing the positive control.

Oxidative stress arises from an imbalance between the production of ROS and the body’s antioxidant defense system. It can result from aerobic metabolism or exposure to chemical stressors. Oxidative stress plays a crucial role in the accumulation of free radicals, contributing to various diseases and accelerating the aging process. Excessive production of free radicals affects processes like apoptosis, cell proliferation, and ion transportation, leading to damage to lipids, proteins, and DNA. This damage has been linked to cardiovascular and neurological diseases, diabetes, liver injury, and even cancer [32]. Rheumatism is closely associated with inflammation, encompassing a group of diseases that affect joints, ligaments, muscles, and other tissues, often accompanied by inflammatory symptoms. Inflammation, a natural response to tissue damage or infection, aims to eliminate pathogens and promote tissue repair. However, in rheumatic diseases, inflammation is typically excessive or prolonged, resulting in chronic inflammation [33]. It is noteworthy that inflammation and oxidative stress are interconnected. Inflammatory responses can trigger the release of more reactive oxygen radicals, exacerbating oxidative stress and creating a feedback loop. The damaging effects of oxidative stress extend to biological molecules such as cell membranes, DNA, and proteins, perpetuating and intensifying inflammatory responses. Phenolics, known for their potent antioxidant properties, have the potential to prevent and treat conditions related to oxidative stress [34]. *L. bulbifera*, abundant in phenolics, exhibits pharmacological effects that can improve the body’s oxidative stress status, thereby promoting blood circulation, lowering blood lipids, and treating rheumatic-related diseases.

Overall, the antioxidant capacity of *L. bulbifera*, particularly its phenolic content, holds great promise in mitigating diseases related to oxidative stress and supporting therapeutic interventions. The wide-ranging scavenging effects observed in this study provide a foundation for further exploration of the potential applications of *L. bulbifera* as a natural source of antioxidants.

Through comprehensive experiments, two important findings have emerged. Firstly, there is a significant disparity between LBR and LBAP in terms of active ingredient content and antioxidant capacity. This suggests that the medicinal efficacy of the whole plant differs substantially from that of the root. Consequently, utilizing the root as the medicament portion proves to be a more advantageous choice. Not only does it ensure treatment efficacy, but it also allows for dosage reduction, optimizing the utilization of the plant’s medicinal properties. Secondly, the choice of extraction solvents plays a crucial role. Our findings reveal that aqueous extracts of LBR consistently exhibit lower active ingredient content and weaker antioxidant capacity compared to alcohol-based solvent extracts. This emphasizes the importance of incorporating an appropriate proportion of ethanol in the extraction process, as it facilitates efficient extraction of active components and enhances antioxidant capacity. *L. bulbifera* has been used as a medicinal plant for centuries, primarily relying on water extraction in traditional methods. However, this method may not fully extract valuable active ingredients, resulting in the waste of medicinal materials. Nevertheless, the efficacy of aqueous extracts remains significant, and the addition of ethanol would undoubtedly yield more favorable results. It is important to note that these suggestions are based on preliminary findings, and further experimental verification is warranted to validate these ideas. In conclusion, considering the disparity in active compound content and antioxidant capacity between LBR and LBAP, as well as the impact of extraction solvents, utilizing LBR as the medicament portions and incorporating ethanol in the extraction process may enhance efficacy. The potential benefits of these approaches should be further studied and validated through rigorous experimentation.

### 2.5. UHPLC-MS Analysis

The methanol extract of LBR exhibited elevated levels of active ingredients and demonstrated exceptional antioxidant activity, warranting further investigation. To unravel the chemical composition of this extract, we employed UHPLC–ESI–Q–TOF–MS analysis. Through this technique, we identified a total of 37 peaks in the UHPLC–MS data (Table S1). To verify the identity of these compounds, we compared them to mass spectral data found in relevant literature, successfully identifying a total of 41 compounds. Please refer to Figure 3 for the structures of the identified compounds. The UHPLC results are presented in Figure 4, visually depict the chromatographic peaks obtained from the analysis. Additionally, for a more comprehensive understanding, the MS and MS/MS results are illustrated in Figures S1–S86. These results highlight that the accurate mass error values for the identified compounds were below 5 ppm [35].

The chemical characterization of the methanol extract of LBR provides valuable insights into the diverse range of compounds present in the extract. Most of the identified compounds have been isolated from LBR and play a significant role in the observed antioxidant activity, contributing to the overall efficacy of LBR as a medicament portion. This detailed analysis serves as a foundation for future research, facilitating the exploration of specific compounds responsible for the observed biological effects and potentially enabling the development of targeted therapeutic interventions.

### 2.6. Stability Studies of Methanol Extract of LBR

**Figure 5:**
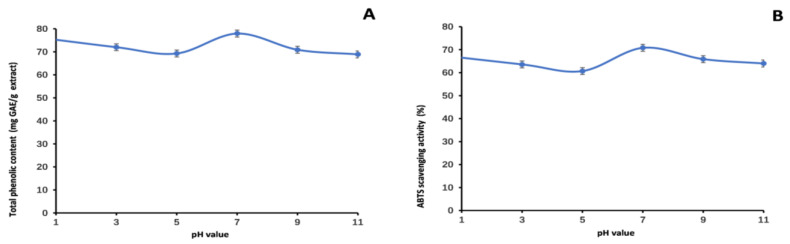
Total phenolic content (**A**) and ABTS (**B**) assays to assess the stability of the methanol extract of *Laportea bulbifera* root at various pH values. (ABTS: 2,2′-Azino-bis (3-ethylbenzothiazoline-6-sulphonic acid) diammonium salt).

**Figure 6:**
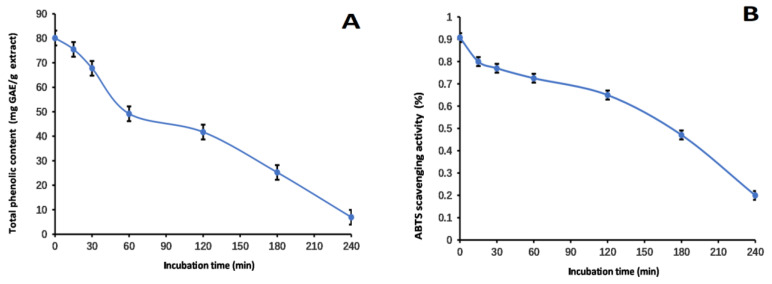
Total phenolic content (**A**) and ABTS (**B**) assays to assess the thermal stability of the methanol extract of *Laportea bulbifera* root. (ABTS: 2,2′-Azino-bis (3-ethylbenzothiazoline-6-sulphonic acid) diammonium salt).

**Figure 7:**
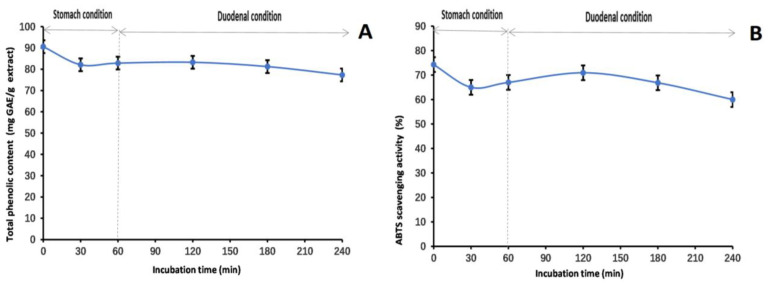
Total phenolic content (**A**) and ABTS (**B**) assays to assess the stability of the methanol extract of *Laportea bulbifera* root an in vitro simulation of the human digestive system. (ABTS: 2,2′-Azino-bis (3-ethylbenzothiazoline-6-sulphonic acid) diammonium salt).

Our research aimed to comprehensively investigate the stability and antioxidant properties of the methanol extract of LBR. The data obtained from Figure 5, Figure 6 and Figure 7 provide valuable insights into the behavior of the extract under different conditions.

Regarding pH stability, when subjected to variations in pH, we observed relatively stable TP_he_C value and ABTS scavenging activity. Interestingly, the highest TP_he_C value was found at pH 7, followed by pH 1. The ABTS scavenging activity exhibited a similar trend. As the alkalinity of the environment increased, the acidic system of the ABTS experiment had a greater impact, which likely contributed to the observed decrease. This underscores the significant influence of pH on the stability of *L. bulbifera* phenolics, indicating that the extract is best stored in neutral or slightly acidic environments.

When investigating the effect of heating time on the extract, a noticeable reduction in both the TP_he_C value and ABTS scavenging activity was observed. This suggests that heat has a substantial impact on the phenolic content of LBR. This experimental finding helps explain why the aqueous extract of LBR has the lowest TP_he_C compared to extractions with solvents such as methanol, ethanol, and 80% ethanol. Water, as a solvent, has a higher reflux temperature of at least 100 °C, while the mentioned alcohols have reflux temperatures below 80 °C. Therefore, incorporating a certain proportion of alcohol as a solvent not only facilitates the extraction of active ingredients but also reduces the reflux temperature, promoting phenolic stability.

To determine the stability of the methanol extract of LBR under physiological conditions, we conducted stability experiments using an in vitro simulation of the human digestive system. Over time, the TP_he_C value gradually decreased, possibly due to the impact of gastric acid, pepsin, trypsin, pancreatin, and bile on the extract. This contributed to the gradual decline in TP_he_C value. The ABTS scavenging activity showed a similar trend. However, it is worth noting that despite these changes, our stability studies revealed that the antioxidant components of the methanol extract of LBR remained stable. This indicates promising potential for maintaining its effectiveness even under various physiological conditions.

Overall, our findings provide valuable insights into the stability and antioxidant properties of the methanol extract of LBR. These findings have implications for its storage, processing, and potential applications in functional foods and pharmaceuticals.

### 2.7. Oxidative Stability Studies of Oils

#### Primarily Oxidation and Polymerization

Frying, which is a popular cooking method, imparts specific sensory characteristics that consumers prefer. However, the high temperatures involved in frying can lead to chemical changes in the oils used, primarily oxidation and polymerization. These changes can negatively affect the shelf life and overall quality of fried food. Synthetic antioxidants are commonly used to enhance the stability of oils, but they come with potential risks and hazards [36]. Therefore, it is crucial to search for safe antioxidants derived from natural sources. One commonly employed approach to assess the primary oxidation state of oils is by evaluating peroxide and acidity values. Additionally, indicators such as K_232_ and K_270_ values are used to monitor primary and secondary lipid oxidation [37].

**Figure 8:**
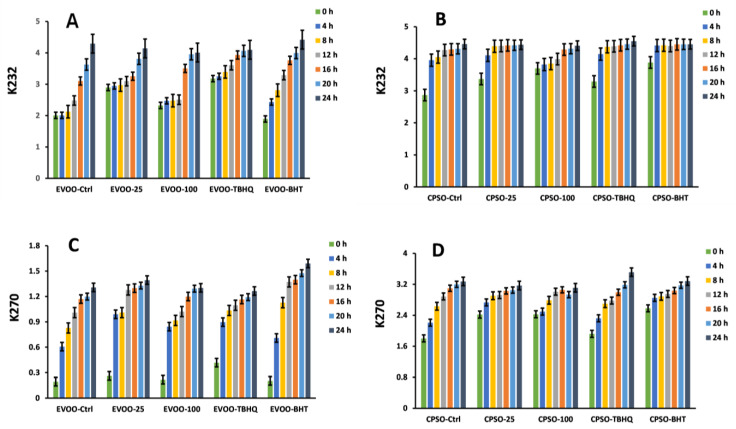
Variations in the levels of conjugated dienes (K_232_) and trienes (K_270_) in extra virgin olive oil (EVOO) (**A**,**C**) and cold-pressed sunflower oil (CPSO) (**B**,**D**) supplemented with the synthetic antioxidants, BHT and TBHQ, and different concentrations of the methanol extract of *Laportea bulbifera* root, at 160 °C (BHT: Butylated hydroxytoluene; TBHQ: tertiary butylhydroquinone).

**Figure 9:**
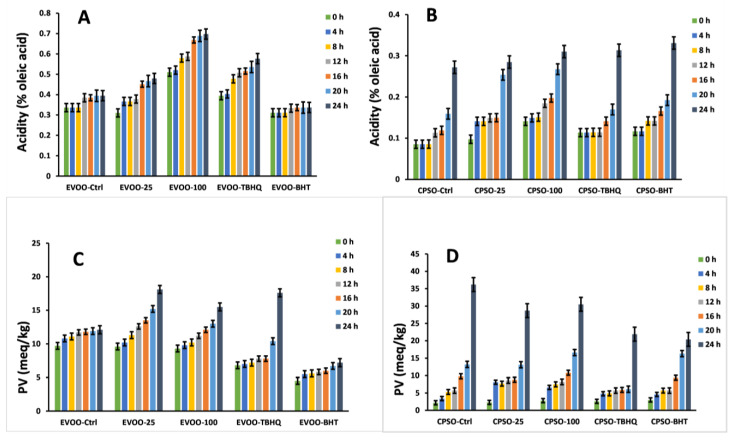
Variations in the levels of acidity values and peroxide values (PV) in extra virgin olive oil (EVOO) (**A**,**C**) and cold-pressed sunflower oil (CPSO) (**B**,**D**) supplemented with the synthetic antioxidants, BHT and TBHQ, and different concentrations of the methanol extract of *Laportea bulbifera* root, at 160 °C (BHT: Butylated hydroxytoluene; TBHQ: tertiary butylhydroquinone).

In the context of evaluating the effectiveness of various antioxidants, our study employed multiple evaluation methods. Remarkably, when testing the impact of adding a significant quantity of LBR methanol extract to extra virgin olive oil (EVOO), we observed a significant antioxidant effect, surpassing that of EVOO-25 and displaying even greater efficacy with EVOO-100 (Figure 8 and Figure 9). Intriguingly, the antioxidant capacity of the methanol extract of LBR proved to be comparable to that of BHT. These results were replicated when using cold-pressed sunflower oil (CPSO) as well. Notably, the dosage of LBR methanol extract utilized in our experiment was merely half that of BHT and tertiary butylhydroquinone (TBHQ), yet it yielded similar outcomes, rendering it a promising natural alternative for stabilizing plant-based oils.

### 2.8. Oral Acute Toxicity Study

In the context of our research, we conducted an oral acute toxicity study to evaluate the safety of the methanol extract of LBR. A group of 20 mice was orally administered a single dose of 2000 mg/kg of the extract. Encouragingly, none of the tested mice exhibited any mortality within a 24 h observation period. This finding suggests that the methanol extract of LBR has a relatively low level of toxicity. *Laportea aestuans*, a plant belonging to the same family as LBR, is reported in the literature to show low toxicity in the brine shrimp lethality test when compound AC isolated from the hexane extract of its leaves is tested [24]. However, there are also reports showing that the volatile oil of its whole plant exhibited toxicity in the same experiment [38]. These experimental results are understandable, as the former is a pure compound test while the latter involves volatile oil. It is normal for the results to differ. Importantly, they use different medicament portions, which is a significant factor affecting the results.

However, it is important to note that these findings do not necessarily guarantee the safety of *Laportea* plants, including *L. bulbifera*, for human consumption. Despite their historical use, there is a lack of comprehensive clinical studies investigating the safety and efficacy of *L. bulbifera* and related species. To establish a comprehensive understanding of their suitability for human use, further research encompassing preclinical and clinical studies is required.

Expanding on the topic, additional studies could focus on examining the potential long-term effects of consuming LBR, particularly in varying doses and durations. Furthermore, it would be beneficial to investigate any potential interactions or contraindications with other medications or underlying medical conditions. By thoroughly assessing the safety and efficacy of LBR through rigorous scientific investigations, we can provide valuable insights into its potential as a viable option for human consumption.

### 2.9. Hepatoprotective Activity

The liver, a vital organ in the human body, performs a multitude of crucial functions. Firstly, it plays a central role in metabolism by regulating the storage, synthesis, and breakdown of carbohydrates, lipids, and proteins. Additionally, the liver is responsible for detoxifying harmful substances, such as drugs and alcohol, through various enzymatic processes. It also produces bile, which is essential for digestion and the absorption of fats. Another key function of the liver is the storage of essential vitamins, minerals, and glycogen, serving as a reservoir for energy and nutrients. Furthermore, it participates in the synthesis of blood-clotting proteins and the removal of old or damaged blood cells. Moreover, the liver aids in maintaining a stable blood glucose level, contributing to overall glucose homeostasis. Overall, the multifaceted functions of the liver are indispensable for maintaining optimal health and well-being [39].

To assess the effectiveness of the methanol extract of LBR in mitigating liver damage, an experimental study was conducted using rats as subjects. A total of forty rats were randomly divided into five groups (*n* = 8), each receiving a different oral treatment. The control group (group I) was given 0.5% carboxymethylcellulose sodium, while the negative control group (group II) received d-galactosamine and 0.5% carboxymethylcellulose sodium. The high-dose group (group III) received the methanol extract of LBR at a dosage of 300 mg/kg BW, and the low-dose group (group IV) received the methanol extract of LBR at a dosage of 150 mg/kg BW. Finally, the comparison group (group V) received silymarin at 100 mg/kg BW, a known liver-protective agent.

**Figure 10:**
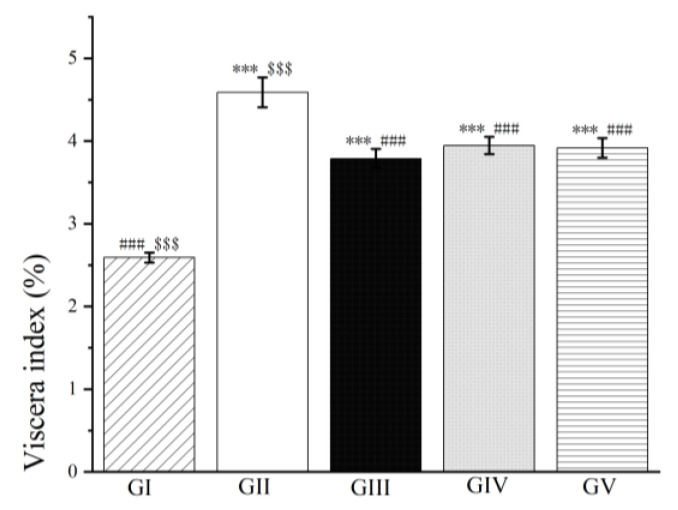
The outcomes of treatment with the methanol extract of LBR on the hepatic viscera index in rats with liver injury.Values are expressed as the mean ± standard error of the mean (*n* = 8). GI: Control group, GII: d-GalN group, GIII: d-GalN + LBR_300_ group, GIV: d-GalN + LBR_150_ group, GV: d-GalN + SMN group. LBR: *Laportea bulbifera* root; d-GalN: d-Galactosamine; SMN: Silymarin. Significantly different from the control group at *** *p* < 0.001. Significantly different from the d-GalN group at ### *p* < 0.001. Significantly different from the d-GalN + SMN group at $$$ *p* < 0.001.

Our findings indicated that pretreatment with both high and low doses of the methanol extract of LBR resulted in a significant reduction in the viscera index of the rats compared to group II. This improvement suggests a notable enhancement in liver condition (*p* < 0.001, see Figure 10). Importantly, there was no significant difference in the protective effect on liver function between the methanol extract of LBR (high and low dose groups) and silymarin (*p* < 0.001, Figure 10).

**Figure 11:**
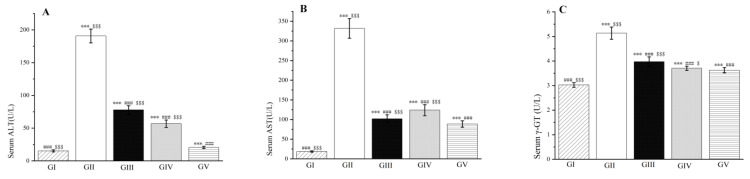
Effects of the methanol extract of LBR on serum ALT (**A**), AST (**B**), and *γ*-GT (**C**) in rats with liver injury. Values are expressed as the mean ± standard error of the mean (*n* = 8). GI: Control group, GII: d-GalN group, GIII: d-GalN + LBR_300_ group, GIV: d-GalN + LBR_150_ group, GV: d-GalN + SMN group. LBR: *Laportea bulbifera* root; d-GalN: d-Galactosamine; SMN: Silymarin; ALT: Alanine aminotransferase; AST: Aspartate aminotransferase; *γ*-GT: *γ*-Glutamyl transpeptidase. Significantly different from the control group at *** *p* < 0.001. Significantly different from the d-GalN group at ### *p* < 0.001. Significantly different from the d-GalN + SMN group at $ *p* < 0.05 and $$$ *p* < 0.001.

Additionally, our study revealed a significant decrease in the activity levels of liver enzymes, including aspartate aminotransferase, alanine aminotransferase, and *γ*-glutamyl transpeptidase, in the groups treated with the methanol extract of LBR compared to group II (*p* < 0.001, as shown in Figure 11). Specifically, compared to group II, the activity levels of alanine aminotransferase decreased by 59% and 70% in groups III and IV, respectively. Similarly, the activity levels of aspartate aminotransferase decreased by 69% and 63%, respectively. Group IV also exhibited a similar effect in reducing *γ*-glutamyl transferase as observed with silymarin. These findings emphasize the potent healing effect of the low-dose methanol extract of LBR on liver damage induced by d-galactosamine. The decrease in liver enzyme activity indicates alleviation of liver injury and inflammation [40]. Therefore, the substantial reduction in the activity of these liver enzymes in the treatment groups provides support for the potential therapeutic application of the methanol extract of LBR in liver-related diseases and disorders. Further research is warranted to elucidate the underlying mechanisms and validate the long-term effectiveness of this natural extract.

**Figure 12:**
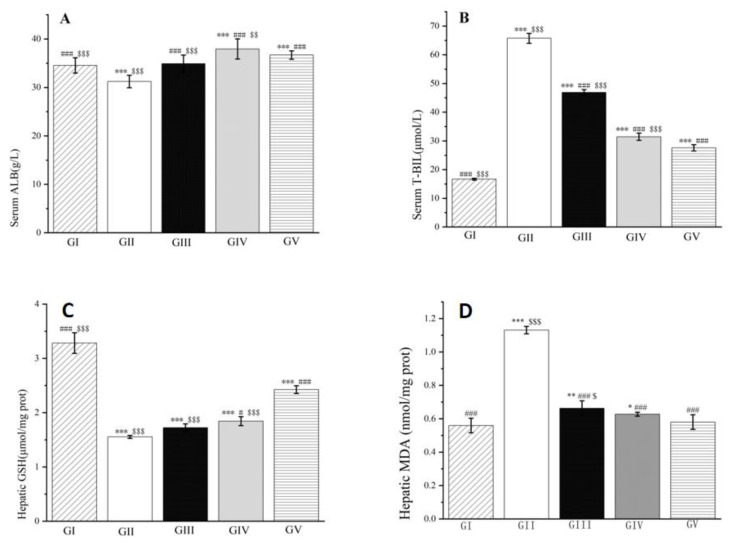
Effects of the methanol extract of LBR on serum ALB (**A**), T-BIL (**B**) and hepatic GSH (**C**), MDA (**D**) in rats with liver injury. Values are expressed as the mean ± standard error of the mean (*n* = 8). GI: Control group, GII: d-GalN group, GIII: d-GalN + LBR_300_ group, GIV: d-GalN + LBR_150_ group, GV: d-GalN + SMN group. LBR: *Laportea bulbifera* root; d-GalN: d-Galactosamine; SMN: Silymarin; ALB: Albumin; T-BIL: Total bilirubin; GSH: Glutathione; MDA: Malondialdehyde. Significantly different from the control group at * *p* < 0.05 ** *p* < 0.01 and *** *p* < 0.001. Significantly different from the d-GalN group at # *p* < 0.05 and ### *p* < 0.001. Significantly different from the d-GalN + SMN group at $ *p* < 0.05 $$ *p* < 0.01 and $$$ *p* < 0.001.

Furthermore, group II rats experienced a significant decrease in albumin levels. However, treatment with both silymarin and the methanol extract of LBR led to a substantial increase in albumin levels after 24 h of modeling (*p* < 0.001). Notably, the low-dose group showed superior efficacy in improving albumin levels compared to the high-dose group. Moreover, following d-galactosamine injection, the concentration of total bilirubin in the blood of rats in group II increased. In contrast, groups III to V, which received the methanol extract of LBR or silymarin, exhibited a remarkable decrease in total bilirubin concentration compared to group II. Interestingly, the low-dose group demonstrated better performance in reducing total bilirubin levels compared to the high-dose group, and its efficacy was comparable to that of silymarin. Once again, the low-dose group outperformed the high-dose group in reducing malondialdehyde levels, and its efficacy was similar to that of silymarin. Additionally, the excessive generation of ROS plays a crucial role in the development of acute liver injury induced by d-galactosamine and diminishes the efficacy of antioxidants in vivo [41]. As a result, malondialdehyde levels increase while glutathione levels decline in the liver of rats. Compared to group II, the methanol extract of LBR exhibited a moderate improvement in glutathione levels and a significant reduction in malondialdehyde levels (*p* < 0.001, as shown in Figure 12). Once again, the low-dose group outperformed the high-dose group in reducing malondialdehyde levels, and its efficacy was similar to that of silymarin.

In summary, our study emphasizes the liver-protective effect of the low-dose methanol extract of LBR, primarily due to its potent antioxidant activity. The overall protective effect of this extract is comparable to that of silymarin, indicating its promising potential as a therapeutic agent for liver-related disorders and diseases. Further research should delve into the underlying mechanisms and conduct additional studies to establish its long-term efficacy and safety profile.

**Figure 13:**
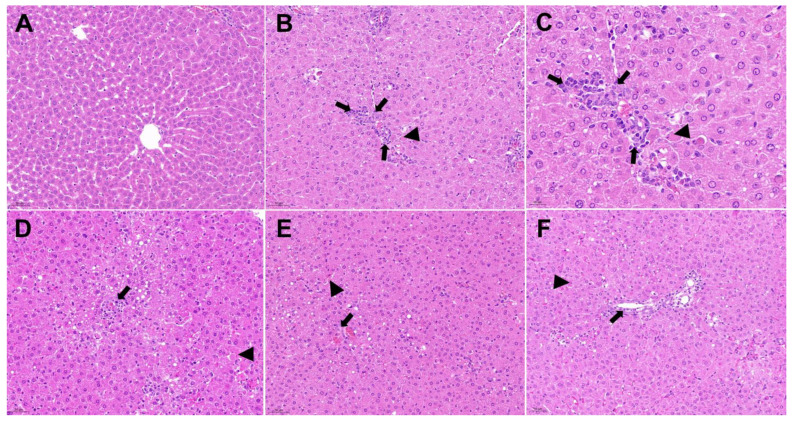
Histological examination of liver sections in different groups (200× magnification). (**A**): Control group (200× magnification); (**B**): d-GalN group (200× magnification); (**C**): d-GalN group (400× magnification); (**D**): d-GalN + LBR_300_ group (200× magnification); (**E**): d-GalN + LBR_150_ group (200× magnification); (**F**): d-GalN + SMN group (200× magnification). LBR: *Laportea bulbifera* root; d-GalN: d-Galactosamine; SMN: Silymarin. No-tailed arrow: single-cell necrosis; Long-tailed arrow: inflammatory cells.

The histopathological findings from our study are presented in Figure 13. In the control group, the liver exhibited a well-defined histological structure, with hepatocytes arranged in a normal pattern and no signs of inflammatory cell infiltration around the portal area (Figure 13A). However, in group II, the liver’s histological structure was disrupted, showing the absence of the hepatic cord and the presence of single-cell necrosis (no-tailed arrow) (Figure 13B), accompanied by a notable presence of inflammatory cells (long-tailed arrow). Upon closer examination at 400× magnification, group II displayed more prominent inflammatory cell infiltration and necrosis (Figure 13C). Remarkably, the high-dose group exhibited significant improvement in hepatocyte injury, with a relatively lower number of inflammatory and necrotic cells compared to group II (Figure 13D). Similarly, the low-dose group demonstrated superior results compared to the high-dose group, showing relatively reduced inflammatory and necrotic cells (Figure 13E). Notably, the positive group exhibited the most significant curative effect, characterized by a significant reduction in inflammatory and necrotic cells, as well as a relatively complete hepatocyte morphology (Figure 13F).

The histopathological examination findings hold great importance in understanding the implications of our study. They provide valuable insights into the extent of liver damage caused by d-galactosamine. The presence of inflammatory cells, necrotic cells, and disrupted histological structure in group II highlights the severity of liver damage caused by d-galactosamine. Conversely, the reduction in these parameters observed in the positive, high-dose, and low-dose groups indicates the hepatoprotective effect of the methanol extract of LBR. These findings further support the potential use of this extract to treat liver disorders. It is worth noting that *Laportea aestuans* has been associated with increased glutathione content and decreased levels of malondialdehyde [42], indicating its beneficial impact on liver health.

## 3. Material and Methods

### 3.1. Materials

*L. bulbifera* was gathered (voucher specimen number: 2021-06-13-002) in Tonghua (latitude N 41°38′1.19′′, longitude E 125°56′1.18′′, altitude 730.7 m, Jilin Province, China) in June 2021. A plant taxonomist (Prof. Junlin. Yu) confirmed the identity of the specimen. The voucher specimen is stored in the Herbarium of Tonghua Normal University.

### 3.2. Methods

#### 3.2.1. Qualitative Phytochemical Analysis

Qualitative phytochemical analysis was performed on 15 types of chemical components, following a previously established method [43]. See the full information in the Supplementary Materials.

#### 3.2.2. Quantitative Phytochemical Analysis

Quantitative phytochemical analysis was conducted to determine the concentration of various compounds such as TCC, TP_ro_C, TP_he_C, TFC, TPAC, TT_an_C, CTC, and GC, using methods previously described in reference [43]. See the full information in the Supplementary Materials.

#### 3.2.3. Antioxidant Activity Assays

Antioxidant activity assays were performed using a range of different methods, including DPPH, ABTS, hydroxyl radicals, superoxide radicals, FRAP, CUPRAC, metal chelating, H_2_O_2_, Singlet oxygen, HClO, and NO. These assays were conducted following previously established protocols [43]. See the full information in the Supplementary Materials.

#### 3.2.4. Stability Studies of Methanol Extract 

pH stability, thermal stability and stability in a gastrointestinal tract model system of methanol extract of LBR were determined in accordance with our previous methods [43]. See the full information in the Supplementary Materials.

#### 3.2.5. Oxidative Stability of Oils

The oxidative stability of EVOO and CPSO was evaluated according to our previous methods [43]. See the full information in the Supplementary Materials.

#### 3.2.6. Oral Acute Toxicity Study

An oral acute toxicity study was conducted according to previously established methods [44]. See the full information in the Supplementary Materials.

#### 3.2.7. Hepatoprotective Experiments

The hepatoprotective experiments were performed, which included animal selection, experimental protocol, histopathological examination, and biochemical analyses, following the procedures outlined in reference [44]. See the full information in the Supplementary Materials.

#### 3.2.8. Statistical Analysis

Statistical analysis was performed to assess the significance of the data. The data were presented as means with the standard error of the mean. One-way analysis of variance with post-hoc least significant difference tests was used to test for significant correlations between groups. Pearson’s correlation analysis was used to investigate the relationship between antioxidant activity and total active constituents. A *p*-value of 0.05, 0.01, and 0.001 were considered significant, highly significant, and very highly significant, respectively. 

## 4. Conclusions

In summary, our study provides valuable insights into *L. bulbifera* and its potential as an ethnomedicine. The qualitative and quantitative analysis of phytochemicals, along with the evaluation of antioxidant properties, shed light on the therapeutic potential of this plant. The methanol extract of LBR, in particular, exhibited excellent stability even under adverse conditions such as different pH values and in vitro digestion, antioxidant capacity, low toxicity and hepatoprotective properties, further emphasizing its potential use in oxidative stress-related diseases.

## Figures and Tables

**Figure 3:**
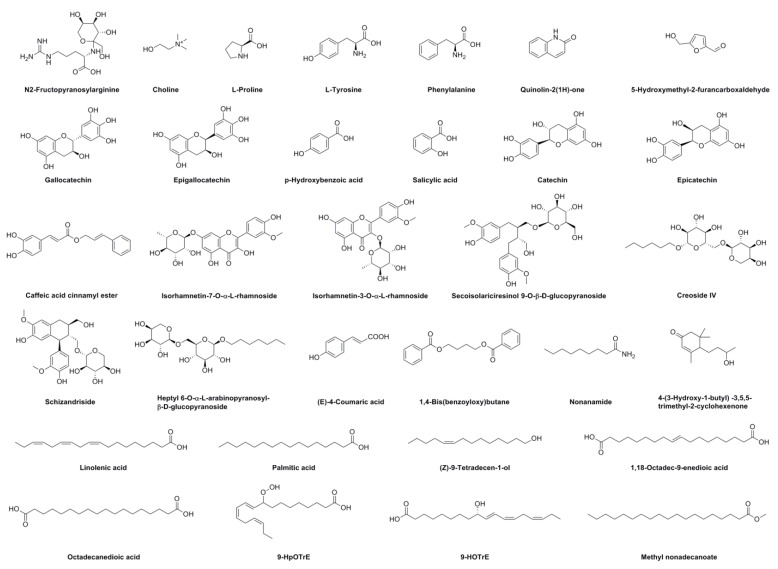
Chemical structures of the compounds identified in methanol extract of *Laportea bulbifera* roots.

**Figure 4:**
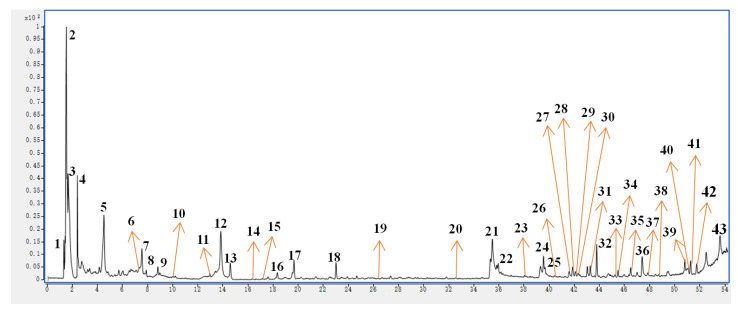
UHPLC–MS results captured in positive-ion mode for methanol extract of *Laportea bulbifera* roots.

**Table 1:** Phytochemical analysis of *Laportea bulbifera* aboveground part and root.

Phytochemical	Type of Test	Sample Solution of LBAP			Sample Solution of LBR		
		Water	Methanol	Petroleum Ether	Water	Methanol	Petroleum Ether
Proteins/amino acids	1. Ninhydrin test	+	○	○	+	○	○
	2. Biuret test	+	○	○	+	○	○
Carbohydrates	1. Fehling’s test	+	○	○	+	○	○
	2. Benedict’s test	+	○	○	+	○	○
	3. Molisch’s test	+	○	○	+	○	○
Phenolics	1. FeCl_3_ test	+	○	○	+	○	○
	2. FeCl_3_-K_3_[Fe(CN)_6_] test	+	○	○	+	○	○
	3. Diazotization test	+	○	○	+	○	○
Organic acids	1. Blue litmus paper test	+	○	○	+	○	○
	2. Bromocresol green test	+	○	○	+	○	○
Tannins	1. FeCl_3_ test	+	○	○	+	○	○
	2. Bromine water test	+	○	○	+	○	○
	3. Lead acetate test	+	○	○	+	○	○
	4. Lime water test	+	○	○	+	○	○
Flavonoids	1. Shinoda test	○	+	○	○	–	○
	2. Alkaline reagent test	○	+	○	○	–	○
	3. AlCl_3_ test	○	+	○	○	–	○
	4. Lead acetate test	○	+	○	○	–	○
Saponins	1. Foam test	–	○	○	–	○	○
Steroids and triterpenoids	1. Liebermann–Burchard test	○	+	○	○	+	○
	2. Salkowski test	○	+	○	○	+	○
Terpenoids	1. CHCl_3_-H_2_SO_4_ test	○	○	–	○	○	–
	2. Vanillin-H_2_SO_4_ test	○	○	–	○	○	–
Alkaloids	1. Bertrad’s reagent	○	+	○	○	+	○
	2. Dragendorff’s reagent	○	+	○	○	+	○
	3. Mayer’s reagent	○	+	○	○	+	○
Anthraquinones	1. Borntrager’s test	○	–	○	○	–	○
	2. Magnesium acetate test	○	–	○	○	–	○
Coumarins and lactones	1. Hydroxamic acid iron test	○	–	○	○	+	○
	2. Diazotization test	○	–	○	○	+	○
	3. Fluorescence test	○	–	○	○	+	○
Volatile oils and fats	1. Phosphomolybdic acid test	○	○	+	○	○	+
	2. Vanillin-H_2_SO_4_ test	○	○	+	○	○	+
	3. Sudan test III	○	○	+	○	○	+
	4. Sudan test IV	○	○	+	○	○	+
Cardiac glycosides	1. Kedde test	○	–	○	○	–	○
	2. Raymond test	○	–	○	○	–	○
	3. Legal test	○	–	○	○	–	○
Cyanogenic glycosides	1. Prussian blue test	–	○	○	–	○	○

(+) indicates presence; (–) indicates absence; (○) indicates no test. LBAP: *Laportea bulbifera* aboveground part. LBR: *Laportea bulbifera* root.

**Table 2:** Extraction yields of *Laportea bulbifera* aboveground part and root extracted with different solvents.

Medicament Portions	Extracting Solvents	Yields (%, *w*/*w*)
Aboveground part	Water	16.60 ± 1.21 ^c,d^
	Methanol	10.40 ± 1.01 ^f,g^
	Ethanol	7.16 ± 0.93 ^g^
	80% Ethanol	12.03 ± 1.00 ^e,f^
Root	Water	14.30 ± 1.29 ^d,e^
	Methanol	24.90 ± 2.03 ^a^
	Ethanol	21.86 ± 2.98 ^a,b^
	80% Ethanol	19.16 ± 2.97 ^b^

^a–g^ Columns with different superscripts indicate a significant difference (*p* < 0.05). Yield was calculated as % yield = (weight of extract/initial weight of dry sample) × 100.

**Table 3:** Total carbohydrate content (TCC), total protein content (TP_ro_C), total phenolic content (TP_he_C), total flavonoid content (TFC), total phenolic acid content (TPAC), total tannin content (TT_an_C), gallotannin content (GC), and condensed tannin content (CTC) of *Laportea bulbifera* extracted with different solvents.

Medicament Portions	Extracting Solvents	TCC (mg GE/g Extract)	TP_ro_C (mg BSAE/g Extract)	TP_he_C (mg GAE/g Extract)	TFC (mg QE/g Extract)	TPAC (mg CAE/g Extract)	TT_an_C (mg TAE/g Extract)	GC (mg GAE/g Extract)	CTC (mg GAE/g Extract)
Aboveground part	Water	118.71 ± 1.48 ^g^	440.23 ± 7.57 ^b^	32.70 ± 0.72 ^f^	4.17 ± 0.18 ^d^	4.85 ± 0.33 ^d^	27.95 ± 0.16 ^g^	12.27 ± 0.03 ^d^	15.09 ± 0.11 ^g^
	Methanol	172.38 ± 3.68 ^e^	N.T.	50.79 ± 0.90 ^e^	69.53 ± 1.25 ^a^	12.22 ± 1.20 ^b^	49.14 ± 0.50 ^e^	NONE	26.63 ± 0.43 ^e^
	Ethanol	98.54 ± 0.51 ^h^	N.T.	23.69 ± 0.23 ^g^	30.65 ± 0.59 ^c^	6.31 ± 0.22 ^c,d^	22.30 ± 0.29 ^h^	7.52 ± 1.87 ^e^	12.40 ± 0.21 ^h^
	80% Ethanol	142.65 ± 1.10 ^f^	N.T.	35.21 ± 0.42 ^f^	58.96 ± 0.30 ^b^	8.46 ± 0.44 ^c^	33.88 ± 0.06 ^f^	12.64 ± 0.26 ^d^	18.70 ± 0.17 ^f^
Root	Water	466.11 ± 4.43 ^d^	2032.86 ± 2.55 ^a^	98.62 ± 0.31 ^d^	NONE	8.46 ± 0.65 ^c^	95.83 ± 0.39 ^d^	44.18 ± 2.06 ^c^	55.23 ± 0.74 ^d^
	Methanol	686.14 ± 3.53 ^a^	N.T.	313.83 ± 4.16 ^a^	NONE	13.85 ± 0.87 ^b^	304.86 ± 3.34 ^a^	58.91 ± 1.10 ^a^	188.70 ± 0.43 ^a^
	Ethanol	505.41 ± 4.57 ^c^	N.T.	302.70 ± 1.79 ^b^	NONE	17.85 ± 3.05 ^a^	268.29 ± 2.69 ^b^	61.27 ± 0.77 ^a^	171.06 ± 0.67 ^b^
	80% Ethanol	641.36 ± 1.46 ^b^	N.T.	272.90 ± 4.00 ^c^	NONE	18.46 ± 2.16 ^a^	246.24 ± 0.83 ^c^	52.55 ± 0.77 ^b^	154.89 ± 1.55 ^c^

^a–h^ Columns with different superscripts indicate a significant difference (*p* < 0.05). GE: Glucose equivalent. BSAE: BSA equivalent. GAE: Gallic acid equivalent. QE: Quercetin equivalent. CAE: caffeic acid equivalent. TAE: Tannic acid equivalent. N.T.: indicates no test. Values are the mean ± standard deviation of three independent experiments.

**Table 4:** Determination of antioxidant activity of various extracts of *Laportea bulbifera* using DPPH, ABTS, hydroxyl and superoxide radicals.

Medicament Portions	Extracting Solvents	DPPH (IC_50_, μg/mL)	ABTS (IC_50_, μg/mL)	Hydroxyl Radicals (IC_50_, μg/mL)	Superoxide Radicals (%, 2143 μg/mL)
Aboveground part	Water	30.96 ± 1.21 ^c^	>500 ^e^	>2500 ^f^	NONE
	Methanol	52.59 ± 4.34 ^e^	42.73 ± 3.77 ^c^	1451.16 ± 46.23 ^e^	20.94 ± 2.02 ^b^
	Ethanol	123.31 ± 0.73 ^f^	25.16 ± 1.80 ^b^	281.49 ± 2.07 ^d^	NONE
	80% Ethanol	43.79 ± 3.81 ^d^	36.65± 5.35 ^b,c^	NONE	NONE
Root	Water	4249.55 ± 265.80 ^g^	222.61 ± 10.50 ^d^	NONE	15.66 ± 4.06 ^b^
	Methanol	2.63 ± 0.11 ^a^	3.00± 0.16 ^a^	176.81 ± 6.71 ^b,c^	19.76 ± 7.78 ^b^
	Ethanol	2.63 ± 0.14 ^a^	2.68 ± 0.15 ^a^	163.20 ± 14.58 ^b^	22.49 ± 1.44 ^b^
	80% Ethanol	3.48 ± 0.06 ^a^	2.97 ± 0.13 ^a^	212.83 ± 8.82 ^c^	16.05 ± 1.08 ^b^
Standard antioxidant	Trolox	2.03 ± 0.13 ^a^	3.78 ± 0.13 ^a^	97.52 ± 1.37 ^a^	N.T.
	BHT	9.34 ± 0.12 ^b^	4.72 ± 0.04 ^a^	208.14 ± 2.53 ^c^	N.T.
	Curcumin	N.T.	N.T.	N.T.	58.23 ± 1.12 ^a^

^a–g^ Columns with different superscripts indicate a significant difference (*p* < 0.05). DPPH: 2,2-Diphenyl-1-picrylhydrazyl. ABTS: 2,2’-Azino-bis (3-ethylbenzothiazoline-6-sulphonic acid) diammonium salt. IC_50_: Concentration required to scavenge 50% of the radicals present in the test solution. Trolox: 6-Hydroxy-2,5,7,8-tetramethylchroman-2-carboxylic acid. BHT: Butylated hydroxytoluene. N.T.: indicates no test. Values are the mean ± standard deviation of three independent experiments.

**Table 5:** Determination of antioxidant activity of different extracts of *Laportea bulbifera* using FRAP, CUPRAC, iron copper chelating.

Medicament Portions	Extracting Solvents	TEAC_FRAP_	TEAC_CUPRAC_	Iron Chelating (IC_50_, μg/mL)	Copper Chelating (IC_50_, μg/mL)
Aboveground part	Water	0.11 ± 0.00 ^g^	NONE	1525.61 ± 88.02 ^c^	430.80 ± 39.49 ^d^
	Methanol	0.14 ± 0.00 ^f^	0.03 ± 0.01 ^d^	1123.73 ± 64.92 ^b^	738.06 ± 45.59 ^f^
	Ethanol	0.16 ± 0.00 ^e^	0.04 ± 0.01 ^d^	1573.85 ± 55.72 ^c^	2027.70 ± 128.13 ^g^
	80% Ethanol	0.16 ± 0.00 ^e^	0.03 ± 0.00 ^d^	1028.78 ± 77.95 ^b^	612.80 ± 1.05 ^e^
Root	Water	0.11 ± 0.00 ^g^	NONE	>2500 ^d^	463.23 ± 56.75 ^d^
	Methanol	0.55 ± 0.01 ^c^	0.87 ± 0.07 ^b^	>2500 ^d^	249.26 ± 10.61 ^c^
	Ethanol	0.63 ± 0.01 ^b^	0.86 ± 0.04 ^b^	>2500 ^d^	144.96 ± 4.38 ^a,b^
	80% Ethanol	0.51 ± 0.01 ^d^	0.69 ± 0.05 ^c^	>2500 ^d^	187.92 ± 6.08 ^b,c^
Standard antioxidant	Trolox	1.00 ± 0.01 ^a^	1.00 ± 0.01 ^a^	N.T.	N.T.
	EDTANa_2_	N.T.	N.T.	2.28 ± 0.11 ^a^	41.58 ± 1.09 ^a^

^a–g^ Columns with different superscripts indicate a significant difference (*p* < 0.05). FRAP: Ferric-reducing antioxidant power. CUPRAC: Cupric ion reducing antioxidant capacity. TEAC: Trolox equivalent antioxidant capacity. EDTANa_2_: Ethylenediaminetetraacetic acid disodium salt. Trolox: 6-Hydroxy-2,5,7,8-tetramethylchroman-2-carboxylic acid. IC_50_: Concentration required to chelate 50% of the ferrous ions present in the test solution. N.T. indicates no test.

**Table 6:** Determination of antioxidant activity of different extracts of *Laportea bulbifera* using H_2_O_2_, singlet oxygen and HClO.

Medicament Portions	Extracting Solvents	H_2_O_2 _ (IC_50_, μg/mL)	Singlet Oxygen (%, 2000 μg/mL)	HClO (IC_50_, μg/mL)
Aboveground part	Water	>2500 ^e^	NONE	NONE
	Methanol	>2500 ^e^	NONE	NONE
	Ethanol	>2500 ^e^	NONE	>990 ^c^
	80% Ethanol	>2500 ^e^	NONE	NONE
Root	Water	1033.64 ± 26.35 ^d^	NONE	80.62 ± 12.67 ^b^
	Methanol	67.74 ± 3.85 ^b,c^	NONE	24.27 ± 0.39 ^a^
	Ethanol	88.21 ± 10.80 ^c^	NONE	21.81 ± 1.22 ^a^
	80% Ethanol	58.27 ± 3.70 ^b^	NONE	14.44 ± 0.92 ^a^
Standard antioxidant	Trolox	N.T.	N.T.	12.81 ± 0.14 ^a^
	Lipoic acid	N.T.	N.T.	26.53 ± 0.80 ^a^
	Gallic acid	20.04 ± 1.07 ^a^	N.T.	N.T.
	Ferulic acid	N.T.	95.3 ± 3.2 ^a^	N.T.

^a–e^ Columns with different superscripts indicate a significant difference (*p* < 0.05). H_2_O_2_: Hydrogen peroxide. HClO: Hypochlorous acid. Trolox: 6-Hydroxy-2,5,7,8-tetramethylchroman-2-carboxylic acid. N.T. indicates no test.

## Data Availability

All data presented in this study are available in the article.

## Supplementary Materials

The following supporting information can be downloaded at: https://www.mdpi.com/article/10.3390/molecules28176256/s1, Table S1: Compounds identified in methanol extract of *Laportea bulbifera* roots. Figures S1–S86: MS spectrum of peak 1–43 and MS/MS spectrum of peak 1–43. References [5,45,46,47,48,49,50,51,52,53,54,55,56,57,58,59,60,61,62,63,64,65,66,67,68] are cited in the Supplementary Materials.

## Sample Availability

Not available.
