# Supplementary material for: Screening the Extract of Laportea bulbifera (Sieb. et Zucc.) Wedd. Based on Active Component Content, Its Antioxidant Capacity and Exploration of Hepatoprotective Activity in Rats
Source: Molecules. 2023 Aug 25;28(17):6256. doi: 10.3390/molecules28176256 (PMC10488916; doi:10.3390/molecules28176256)
Supplement: Supplementary file 1 [file molecules-28-06256-s001.zip › molecules-2565765-supplementary.docx]

**Supplementary Material**

Screening the Extract of *Laportea bulbifera* (Sieb. et Zucc.) Wedd. Based on Active Component Content, Its Antioxidant Capacity and Exploration of Hepatoprotective Activity in Rats

Jiaxin Feng ^1,2,†^, Yue Sun ^1,2,†^, Zhongbao Wei ^3^, Hui Sun ^2^, Li Li ^1,2^, Junyi Zhu ^2,4^, Guangqing Xia ^1,2,4^
and Hao Zang ^1,2,4,^*

^1^ College of Pharmacy, Yanbian University, Yanji 133000, China; 13630304082@163.com (J.F.); 15981309048@163.com (Y.S.); lili1984@thnu.edu.cn (L.L.); qingguangx@thnu.edu.cn (G.X.)

^2^ Green Medicinal Chemistry Laboratory, School of Pharmacy and Medicine, Tonghua Normal University, Tonghua 134002, China; sunhui9405@163.com (H.S.); swx0527@163.com (J.Z.)

^3^ Institute of Scientific and Technical Information of Jilin, Changchun 130033, China; 13894531050@163.com

^4^ Key Laboratory of Evaluation and Application of Changbai Mountain Biological Gerplasm Resources of Jilin Province, Tonghua 134002, China

***** Correspondence: zanghao2013@thnu.edu.cn; Tel.: +86-435-320-2678

† These authors contributed equally to this work.

Qualitative phytochemical analysis

Tests for proteins

Ninhydrin test

Mix 1 mL of the aqueous extraction solution with 1 mL of 0.2% ninhydrin solution. Boil the mixture for 5 min. The presence of amino acids or proteins is indicated by the development of a purple color.

Biuret test

Combine 1 mL of the aqueous extraction solution with 1 mL of solution A (0.1 g/mL NaOH). Add two drops of solution B (0.01 g/mL CuSO_4_). Shake the mixture. The presence of amino acids or proteins is indicated by a purple, red, or purplish-red color.

Tests for carbohydrates

Fehling’s test

Mix equal volumes of solution A (34.66 g of CuSO_4_·5H_2_O dissolved in 500 mL of distilled water) and solution B (173 g of sodium potassium tartrate tetrahydrate and 50 g of NaOH dissolved in 500 mL of distilled water). Mix 1 mL of this mixture with 1 mL of the aqueous extraction solution. Gently boil the resulting mixture. The presence of reducing sugars is indicated by the formation of a brick-red precipitate.

Benedict’s test

Mix 1 mL of the aqueous extraction solution with 1 mL of Benedict's reagent. Gently boil the resulting mixture. The presence of carbohydrates is indicated by the formation of a reddish-brown precipitate.

Molisch’s test

Mix 1 mL of the aqueous extraction solution with 1 mL of Molisch's solution (2 g of *α*-naphthol dissolved in 100 mL of 95% ethanol). Carefully pour the mixture into another test tube containing 1 mL of H_2_SO_4_. The presence of carbohydrates is indicated by the formation of a purple ring at the interface of the aqueous phase and the organic phase.

Tests for phenolics

FeCl_3_ test

Mix 1 mL of the aqueous extraction solution with 1 mL of 2% FeCl_3_ solution. The presence of phenolics is indicated by the development of a blue-green or black color.

FeCl_3_-K_3_[Fe(CN)_6_] test

Apply a few drops of the aqueous extraction solution onto a thin-layer chromatography plate. Spray the chromogenic reagent (a mixture of 1% K_3_[Fe(CN)_6_] solution and 2% FeCl_3_ solution in equal volumes) onto the plate. This will generate a blue color. Afterwards, spray 2 M HCl onto the plate. A darker color indicates the presence of phenolics.

Diazotization test

Mix 1 mL of the aqueous extraction solution with 1 mL of 3% Na_2_CO_3_ solution. Boil the resulting mixture for 3 min and then cool it in ice water. Add two drops of freshly prepared diazotization reagent. The presence of phenolics is indicated by the development of a red color.

Tests for organic acids

Blue litmus paper test

Place a few drops of the aqueous extraction solution onto a blue litmus paper. The presence of organic acids is indicated by the development of a red color.

Bromocresol green test

Add a few drops of the aqueous extraction solution onto a thin-layer chromatography plate. Spray the chromogenic reagent (0.1 g of bromocresol green dissolved in 500 mL of ethanol, mixed with 5 mL of 0.1 N NaOH) onto the plate. The presence of organic acids is indicated by the development of a yellow color on a blue background.

Tests for tannins

FeCl_3_ test

Follow the same experimental procedure as described in the FeCl_3_ tests. The presence of tannins is indicated by the development of a blue-green or black color.

Bromine water test

Add bromine water (3%) to 1 mL of the aqueous extraction solution. The presence of tannins is indicated by the formation of a precipitate.

Lead acetate test

Add 1 mL of lead acetate solution to 1 mL of the aqueous extraction solution. The formation of a precipitate is considered evidence for the presence of tannins.

Lime water test

Add clear lime water (1 mL) to 1 mL of the aqueous extraction solution. The presence of tannins is indicated by the formation of a precipitate.

Tests for flavonoids

Shinoda test

Add an appropriate amount of magnesium powder to 1 mL of the methanol extraction solution, followed by two drops of HCl. The presence of flavonoids is indicated by the development of a red to red-purple color.

Alkaline reagent test

Combine 1 mL of methanol extraction solution with 1 mL of 2% NaOH solution. The presence of flavonoids is indicated by the initial development of an intense yellow color, which changes to colorless upon the addition of a few drops of diluted HCl.

AlCl_3_ test

Add a few drops of the methanol extraction solution onto a thin-layer chromatography plate. Then, spray a 1% AlCl_3_ methanol solution onto the plate. The presence of flavonoids is indicated by the observation of yellow-green fluorescence under an ultraviolet lamp.

Lead acetate test

Introduce a few drops of lead acetate solution to 1 mL of the methanol extraction solution. The presence of flavonoids is indicated by the formation of a yellow precipitate.

Tests for saponins

Foam test

Combine 1 mL of the aqueous extraction solution with 5 mL of distilled water. Shake the mixture and allow it to stand for 10 min. The presence of saponins is indicated by the formation of a stable foam.

Tests for steroids and triterpenoids

Liebermann-Burchard test

Place 5 mL of the aqueous extraction solution in an evaporating dish and evaporate it. Dissolve the residue in 1 mL of acetic anhydride, followed by the addition of one drop of H_2_SO_4_. The development of a red or purple color indicates the presence of triterpenoids, while a blue-green color indicates the presence of steroids.

Salkowski test

Mix 1 mL of the methanol extraction solution with 1 mL of CHCl_3_. Carefully add 1 mL of H_2_SO_4_ and gently shake the mixture. The presence of steroids or triterpenoids is indicated by a reddish-brown color in the CHCl_3_ layer and green fluorescence in the H_2_SO_4_ layer.

Tests for terpenoids

CHCl_3_-H_2_SO_4_ test

Mix 1 mL of the methanol extraction solution with 2 mL of CHCl_3_ and evaporate the mixture. Carefully add 2 mL of H_2_SO_4_ and heat the mixture at 60 °C for 2 min. The development of a grey color indicates the presence of terpenoids.

Vanillin-H_2_SO_4_ test

Add a few drops of the petroleum ether extraction solution onto a thin-layer chromatography plate. Prepare a chromogenic reagent by dissolving 5 g of vanillin in 100 mL of 10% H_2_SO_4_ ethanol solution and spray it onto the plate. The presence of volatile oils, terpenoids, and steroids is indicated by the development of a red, blue, or purple color.

Tests for alkaloids

Bertrad’s reagent test

Mix 1 mL of the methanol extraction solution with 1 mL of tungstosilicic acid reagent. The reagent is prepared by dissolving 5 g of tungstosilicic acid hydrate in 100 mL of distilled water and adjusting the pH to 2.0 by adding a small amount of HCl. The presence of alkaloids is indicated by the formation of a pale yellow or off-white precipitate.

Dragendorff’s reagent test

Combine 1 mL of the ethanol extraction solution with 1 mL of Dragendorff's reagent. To prepare the reagent, mix equal volumes of solution A (850 mg of bismuth subnitrate dissolved in 40 mL of distilled water and 10 mL of acetic acid) and solution B (8 g of KI dissolved in 20 mL of distilled water) to create a stock solution. Take a sample of the stock solution (10 mL) and mix it with 20 mL of acetic acid, then dilute to 100 mL with distilled water. The formation of a light yellow or reddish-brown precipitate indicates the presence of alkaloids.

Mayer’s reagent test

Mix 1 mL of the ethanol extraction solution with 1 mL of Mayer’s reagent. To prepare the reagent, combine solution A (1358 mg of HgCl_2_ dissolved in 60 mL of distilled water) and solution B (5 g of KI dissolved in 10 mL of distilled water), then dilute to 100 mL with distilled water. The presence of alkaloids is indicated by the formation of a white or light yellow precipitate.

Tests for anthraquinones

Borntrager’s test

Mix 1 mL of the methanol extraction solution with 1 mL of 10% NaOH solution. Observe the development of a red color. Then, add a small volume of 30% H_2_O_2_ solution and heat the mixture at 60 °C. The addition of HCl solution will cause the red color to disappear, followed by the addition of NaOH solution, resulting in the development of a red color indicating the presence of anthraquinones.

Magnesium acetate test

Add three drops of 1% magnesium acetate methanol solution to 1 mL of the methanol extraction solution. The presence of anthraquinones is indicated by the development of a red color.

Tests for coumarins and lactones

Hydroxamic acid iron test

Add 3 drops of a 7% hydroxylamine hydrochloride methanol solution and a 10% KOH methanol solution to 1 mL of methanol extraction solution. Heat the mixture at 60 °C and adjust the pH to 3.0-4.0 by adding 5% HCl. Then, add 2 drops of a 1% FeCl_3_ ethanol solution. The presence of coumarins and lactones is indicated by the development of an orange or purple color.

Diazotization test

Use methanol extraction solution and follow the same experimental procedure as described in Diazotization test. The presence of coumarins and lactones is indicated by the development of a red color.

Fluorescence test

Add a few drops of methanol extraction solution onto a thin-layer chromatography plate and observe for blue-green fluorescence under an ultraviolet lamp. Spray the plate with a 1% KOH solution. The presence of coumarins is indicated by the generation of intense fluorescence.

Tests for volatile oils and fats

Phosphomolybdic acid test

Add a few drops of petroleum ether extraction solution onto a thin-layer chromatography plate and spray with a 25% phosphomolybdic acid solution (2.5 g of phosphomolybdic acid hydrate dissolved in 10 mL of absolute ethanol). The presence of lipids, triterpenoids, and steroids is indicated by the development of a blue color.

Vanillin-H_2_SO_4_ test

Use methanol extraction solution following the same procedure as described in vanillin-H_2_SO_4_ test. The presence of volatile oils, terpenoids, and steroids is indicated by the development of a red, blue, or purple color.

Sudan test

Add 1 mL of methanol extraction solution to which one drop of sudan III solution (0.1 g of sudan III dissolved in 10 mL of 95% ethanol) is added. The presence of oils and fats is indicated by the development of an orange color. Similarly, add 1 mL of methanol extraction solution to which one drop of sudan IV solution (0.01 g of sudan IV dissolved in 5 mL of acetone, followed by the addition of 5 mL of 70% ethanol) is added. The presence of oils and fats is indicated by the development of a red color.

Tests for cardiac glycosides

Kedde test

Add a thin-layer chromatography plate and a few drops of methanol extraction solution. Prepare a chromogenic reagent by mixing equal volumes of solution A (2% methanol solution of 3,5-dinitrobenzoic acid) and solution B (2 M KOH solution). Spray the plate with the reagent. The presence of cardiac glycosides is indicated by the development of a purple-red color, followed by a change to colorless.

Raymond test

Dissolve 1 mg of the methanol extract in 50% ethanol. Add 0.1 mL of a 2% *m*-dinitrobenzene ethanol solution and 0.2 mL of a 20% NaOH solution. The presence of cardiac glycosides is indicated by the development of a blue-purple color.

Legal test

Dissolve 1 mg of the methanol extract in two drops of pyridine. Add one drop of a 3% sodium nitroprusside solution and one drop of a 2 M NaOH solution. The presence of cardiac glycosides is indicated by the development of a dark red color, followed by a change to colorless.

Tests for cyanogenic glycosides

Prussian blue test

Place 1 g of LBAP or LBR powder in a test tube and add 2 mL of distilled water, immediately wrapping the test tube with filter paper. Add one drop of a 10% KOH solution onto the filter paper, then heat the system at 60 °C for 30 min. Sequentially add one drop each of a 10% ferrous sulphate, 10% HCl, and 5% FeCl_3_ onto the filter paper. The presence of cyanogenic glycosides is indicated by a blue color on the filter paper.

Quantitative phytochemical analysis

Determination of TCC

Briefly, 250 μL of *L. bulbifera* extract in distilled water, 125 μL of phenol solution (5%), and 625 μL of H_2_SO_4_ were mixed in an Eppendorf tube and incubated for 30 min. Subsequently, 200 μL of the sample was pipetted from each Eppendorf tube onto a microplate. A calibration curve was produced based on glucose (0–200 mg/L) as a standard. The absorbance of the sample was recorded at 490 nm against a blank sample consisting of *L. bulbifera* extract with distilled water. The mean of three readings was used and TCC was expressed in mg GE/g of *L. bulbifera* extract.

Determination of TP_ro_C

Briefly, 200 μL of bicinchoninic acid working solution and 20 μL of *L. bulbifera* extract in distilled water were mixed in a microplate and incubated at 37 ^o^C for 30 min. A calibration curve was produced based on bovine serum albumin (0–500 mg/L) as a standard. The absorbance of the sample was recorded at 562 nm against a blank sample consisting of *L. bulbifera* extract with distilled water. The mean of three readings was used and TP_ro_C was expressed in mg BSAE/g of *L. bulbifera* extract.

Determination of TP_he_C

Briefly, 100 μL of Folin & Ciocalteu’s phenol reagent (FC reagent) (1 M) and 200 μL of *L. bulbifera* extract in distilled water were mixed in an Eppendorf tube and incubated for 5 min. Subsequently, 500 μL of Na_2_CO_3_ solution (20%) was added and allowed to stand at room temperature for 40 min in the dark (with mixing every 10 min). Subsequently, 200 μL of the sample was pipetted from each Eppendorf tube onto a microplate. A calibration curve was produced based on gallic acid (0–100 mg/L) as a standard. The absorbance of the sample was recorded at 750 nm against a blank sample consisting of *L. bulbifera* extract with distilled water and Na_2_CO_3_. The mean of three readings was used and TP_he_C was expressed in mg GAE/g of *L. bulbifera* extract.

Determination of TFC

Briefly, 100 μL of AlCl_3_ (2%) in methanol and 100 μL of *L. bulbifera* extract in methanol were mixed in a microplate and incubated at room temperature for 10 min. A calibration curve was produced based on quercetin (0–100 mg/L) as a standard. The absorbance of the sample was recorded at 415 nm against a blank sample consisting of *L. bulbifera* extract with methanol. The mean of three readings was used and TFC was expressed in mg QE/g of *L. bulbifera* extract.

Determination of TPAC

Briefly, 20 μL of *L. bulbifera* extract in distilled water, 20 µL of Arnow reagent, 20 µL of HCl solution (0.1 M), 120 µL of distilled water and 20 µL of NaOH solution (1 M) were mixed in a microplate and recorded immediately at 490 nm against a blank sample (Arnow reagent was replaced with distilled water). A calibration curve was produced based on caffeic acid (0–100 mg/L) as a standard. The mean of three readings was used and TPAC was expressed in mg CAE/g of *L. bulbifera* extract.

Determination of TT_an_C

Briefly, 200 μL of FC reagent (1 M) and 200 μL of *L. bulbifera* extract in distilled water were mixed in an Eppendorf tube and incubated for 5 min. Subsequently, 100 μL of Na_2_CO_3_ solution (20%) and 1500 μL of distilled water were added and allowed to stand at room temperature for 30 min in the dark (with mixing every 10 min). Subsequently, 200 μL of the sample was pipetted from each Eppendorf tube onto a microplate. A calibration curve was produced based on tannic acid (0–200 mg/L) as a standard. The absorbance of the sample was recorded at 725 nm against a blank sample consisting of *L. bulbifera* extract with distilled water and Na_2_CO_3_. The mean of three readings was used and TT_an_C was expressed in mg TAE/g of *L. bulbifera* extract.

Determination of GC

Briefly, 875 µL of *L. bulbifera* extract in methanol and 375 µL of saturated KIO_3_ solution were mixed in an Eppendorf tube and incubated at 15 ^o^C for 120 min. A calibration curve was produced based on gallic acid (0–400 mg/L) as a standard. The absorbance of the sample was recorded at 550 nm against a blank sample (KIO_3_ was replaced with distilled water). The mean of three readings was used and GC was expressed in mg GAE/g of *L. bulbifera* extract.

Determination of CTC

Briefly, 4 mg of phloroglucinol was added to 2 mL of *L. bulbifera* extract in distilled water. Subsequently, 1 mL of HCl solution and 1 mL of formaldehyde solution were added and mixed in an Eppendorf tube and incubated at room temperature overnight. The precipitate was separated by filtration, the unprecipitated phenolics were measured in the filtrate according to the method of TP_he_C.

Antioxidant activity assays

DPPH assay

Briefly, 100 µL of L. bulbifera extract in methanol and 100 µL of DPPH in methanol (50 µM) were mixed in a microplate and allowed to stand at room temperature for 20 min in the dark. The absorbance of the sample was recorded at 515 nm. The Half-maximal inhibitory concentration (IC_50_) values were calculated and expressed as the mean ± standard deviation (SD) in μg/mL.

ABTS assay

Briefly, 190 μL of diluted ABTS solution and 10 μL of *L. bulbifera* extract in DMSO were mixed in a microplate and incubated for 20 min in the dark. The absorbance of the sample was recorded at 734 nm. The IC_50_ values were calculated and expressed as the mean ± SD in μg/mL.

Hydroxyl radical assay

Briefly, 50 µL of *L. bulbifera* extract in DMSO, 50 µL of FeSO_4_ solution (3 mM) and 50 µL of H_2_O_2_ solution (3 mM) were mixed in a microplate and incubated for 10 min. After then 50 µL of salicylic acid solution (6 mM) was added and incubated at room temperature for 30 min in the dark. The absorbance of the sample was recorded at 492 nm. The IC_50_ values were calculated and expressed as the mean ± SD in μg/mL.

Superoxide radical assay

Briefly, 45 µL of *L. bulbifera* extract in DMSO (10 mg/mL), 15 µL of *p*-nitroblue tetrazolium chloride (NBT) in DMSO (1 mg/mL) and 150 µL of NaOH in DMSO (50 μM) were mixed in a microplate and the absorbance of the sample was recorded immediately at 560 nm against a blank sample (NBT was replaced with DMSO). The scavenging activity was expressed as % scavenging rate and was calculated as follows:

$$\%scavenging=\left(1-\frac{\Delta A_{sample}}{\Delta A_{control}}\right)\times100\%$$

FRAP assay

Briefly, 20 µL of *L. bulbifera* extract in DMSO and 180 µL of FRAP reagent were mixed in a microplate and incubated at 37 ^o^C for 30 min in the dark. A calibration curve was produced based on FeSO_4_ (0–600 mg/L) as a standard. The absorbance of the sample was recorded at 595 nm. Trolox was used as positive reference. The FRAP was expressed as the Trolox Equivalent Antioxidant Capacity (TEAC_FRAP_).

CUPRAC assay

Briefly, 20 µL of CuCl_2_ solution (100 mM), 50 µL of neocuproine in 96% ethanol (7.5 mM), 50 µL of NH_4_Ac solution, 20 µL of L. bulbifera extract in DMSO, and 30 µL of distilled water were mixed in a microplate and incubated at 50 ^o^C for 20 min. This mixture was allowed to stand at room temperature for 10 min. The absorbance of the sample was recorded at 450 nm. The CUPRAC was expressed as the Trolox Equivalent Antioxidant Capacity (TEAC_CUPRAC_).

Iron chelating assay

Briefly, 50 µL of L. bulbifera extract in methanol, 110 µL of ultra-pure water, and 20 µL of FeCl_2_ solution (0.5 mM) were mixed in a microplate and incubated for 5 min. Subsequently, 20 µL of ferrozine solution (2.5 mM) was added and incubated for 10 min. The absorbance was recorded at 562 nm against a blank sample (ferrozine solution was replaced with water). The IC_50_ values were calculated and expressed as the mean ± SD in μg/mL.

Copper chelating assay

Briefly, 40 µL of L. bulbifera extract in ultra-pure water, 140 µL of acetic acid-sodium acetate buffer solution (pH 6.0, 50 mM), and 10 µL of CuSO_4_ solution (5 mM) were mixed in a microplate and incubated for 30 min. Subsequently, 10 µL of pyrocatechol violet solution (4 mM) was added and incubated for 30 min. The absorbance was recorded at 632 nm against a blank sample (pyrocatechol violet was replaced with water). The IC_50_ values were calculated and expressed as the mean ± SD in μg/mL.

H_2_O_2_ assay

Briefly, 70 µL of phenol solution (pH 7.0, 12 mM, in 84 mM phosphate buffer (PBS)), 20 µL of 4-aminoantipyrine solution (pH 7.0, 0.5 mM, in 84 mM PBS), 32 μL of H_2_O_2_ solution (pH 7.0, 0.7 mM, in 84 mM PBS), 8 µL of horseradish peroxidise (EC 1.11.1.7) solution (pH 7.0, 1 U/mL, in 84 mM PBS) and 70 µL of L. bulbifera extract (pH 7.0, in 84 mM PBS) were mixed in a microplate and the absorbance of the sample was recorded immediately at 504 nm against a blank sample (phenol solution was replaced with PBS). The IC_50_ values were calculated and expressed as the mean ± SD in μg/mL.

Singlet oxygen assay

Briefly, 40 µL of L. bulbifera extract (pH 7.4, in 45 mM PBS), 50 µL of N,N-Dimethyl-4-nitrosoaniline (pH 7.4, 0.2 mM, in 45 mM PBS), 20 μL of histidine solution (pH 7.4, 0.1 mM, in 45 mM PBS), 20 µL of NaClO solution (pH 7.4, 0.1 mM, in 45 mM PBS), 20 μL of H_2_O_2_ (pH 7.4, 0.1 mM, in 45 mM PBS) and 50 µL of PBS (pH 7.4, 45 mM) were mixed in a microplate and allowed to stand at room temperature for 40 min. The absorbance of the sample was recorded at 440 nm against a blank sample (L. bulbifera extract was replaced with PBS). The IC_50_ values were calculated and expressed as the mean ± SD in μg/mL.

HClO assay

HClO was freshly prepared by adjusting the pH of a 1% (v/v) of NaClO to 6.2 with 1% H_2_SO_4_. The concentration of HClO was determined by reading the absorbance at 235 nm and using the molar extinction coefficient of 100 M^-1^ cm^-1^. Briefly, twenty-microliters of L. bulbifera extract aqueous solution, 20 µL of 150 mM taurine aqueous solution, 20 µL of 0.5 mM HClO solution and 140 μL of PBS (pH 7.4, 50 mM) were mixed in a microplate and incubated for 10 min. Subsequently, 2 µL of 2 M KI aqueous solution was added and mixed. The absorbance was recorded at 350 nm against a blank sample (taurine and HClO were replaced with water). IC_50_ values were calculated and expressed as the mean ± SD in μg/mL.

NO assay

Briefly, 3 mL of L. bulbifera extract in methanol (1 mg/mL) and 3 mL of sodium nitroprusside solution (pH 7.4, 5 mM, in 0.1 M PBS) were mixed in an Eppendorf tube and incubated at 25 ^o^C for 150 min. At intervals, 100 μL of the sample was pipetted from each Eppendorf tube onto a microplate containing 100 µL of Griess reagent. In the control group, L. bulbifera extract was replaced with methanol. The absorbance was recorded at 546 nm against a blank sample (Griess reagent was replaced with distilled water).

Reagents and Chemicals

*p*-Nitroblue tetrazolium chloride (NBT) was purchased from Sigma-Aldrich. Curcumin, salicylic acid, *L*-ascorbic acid, 2,4,6-tri(2-pyridyl)-s-triazine (TPTZ), ammonium acetate (NH_4_Ac), cupric sulphate, ferrous sulfate heptahydrate (FeSO_4_·7H_2_O), copper sulphate (CuSO_4_), taurine, 4-aminoantipyrine, lipoic acid, ferulic acid, sulfanilamide, cupric chloride dihydrate (CuCl_2_·2H_2_O), phosphoric acid (H_3_PO_4_), ninhydrin hydrate, quercetin, naphthylethylenediamine dihydrochloride, D-(+)-glucose, butylated hydroxytoluene (BHT), 2,9-dimethyl-1,10-phenanthroline (Neocuproine, Nc), *α*-naphthol, iodine, tertiary butylhydroquinone (TBHQ), 3,5-dinitrosalicylic acid (DNS), gelatin, potassium iodide (KI), ferric chloride (FeCl_3_), 4-nitroaniline, sodium nitrite, antimony trichloride, calcium hydroxide (Ca(OH)_2_), ABTS, copper sulfate pentahydrate (CuSO_4_·5H_2_O), phosphomolybdic acid hydrate, hydroxylamine hydrochloride, potassium hydroxide, vanillin, 3,5-dinitrobenzoic acid, phenol, dipotassium hydrogen phosphate, potassium dihydrogen phosphate, sodium dihydrogen phosphate, dibasic sodium phosphate, sodium nitroprusside dehydrate, sodium hypochlorite (NaClO) (10% active chloride), tannic acid, potassium persulfate, potassium chloride (KCl), sodium acetate, gallic acid, sodium molybdate, arbutin, *L*-tyrosine, urea, phloroglucinol, potassium iodate were purchased from Energy Chemical. Benedict’s Reagent was purchased from Adamas. DPPH was purchased from Alfa Aesar. Bromocresol green, trolox, pyrocatechol violet, sudan III, and sudan IV were purchased from TCI. Folin & Ciocalteu’s phenol reagent (FC reagent), aluminum chloride hexahydrate (AlCl_3_·6H_2_O), linoleic acid, 3-(2-pyridyl)-5,6-diphenyl-1,2,4-triazine-4′,4″-disulfonic acid sodium salt (Ferrozine), ferrous chloride tetrahydrate (FeCl_2_·4H_2_O), sodium potassium tartrate tetrahydrate (Rochelle salt), ethylenediaminetetraacetic acid disodium salt dihydrate (EDTANa_2_·2H_2_O), potassium ferricyanide (K_3_[Fe(CN)_6_]), Lead(II) acetate trihydrate, tungstosilicic acid hydrate, bismuth subnitrate, mercury(II) chloride (HgCl_2_), pepsin (32 U/mg), pancreatin, bovine bile extract, magnesium acetate, sodium thiosulfate standard solution (0.1 M), potassium hydroxide standard solution (0.1 M), phenolphthalein, tween 40, and 1,3-dinitrobenzene were purchased from Xiya Reagent. Concentrated sulfuric acid (H_2_SO_4_), phenol, sodium carbonate (Na_2_CO_3_), methanol, ethanol, acetone, ethyl acetate, dichloromethane, hexane, dimethyl sulfoxide (DMSO), petroleum ether (60–90 °C), sodium hydroxide (NaOH), concentrated hydrochloric acid (HCl), sodium chloride (NaCl), magnesium powder, acetic acid, ammonium hydroxide (NH_3_·H_2_O), acetic anhydride, 30% hydrogen peroxide (H_2_O_2_), formaldehyde, and 3% bromine water were purchased from Sinopharm. All reagents and solvents used were analytical grade. Litmus paper blue was purchased from Tianjin Jinda Chemical Reagent Co., Ltd. BCA kit was purchased from Beyotime. Trypsin (2500 U/mg) was purchased from Aladdin (Bay City, MI, USA).

*Preparation of Laportea bulbifera extracts for quantitative phytochemical analysis and ultra-high performance liquid chromatography-mass spectrometry (UHPLC-MS) analysis*

The collected samples of *Laportea bulbifera* were divided into aboveground parts and roots, both of them were dried in a cool ventilated place, and pulverized to powder separately. The powder (20 g) of *Laportea bulbifera* aboveground part (LBAP) or *Laportea bulbifera* root (LBR) was added to a single-neck round-bottomed flask (glass, 500 mL), followed by addition of 200 mL of various solvents (water, methanol, ethanol or 80% ethanol) and refluxing using a hotplate magnetic stirrer employing methyl silicone oil as the heating medium for 6 h at the respective boiling points of the solvents. Extracts were filtered through a Whatman No.1 filter paper and evaporated under reduced pressure at <50 °C until dry using a rotary evaporator. All dried extracts were weighed and stored at −20 °C until use. Yield was calculated as % yield = (weight of dry extract/initial weight of dry sample) × 100.

*Preparation of preliminary experimental solutions for qualitative phytochemical analysis*

Aqueous extraction solutions

The powder (5 g) of LBAP or LBR was weighed and passed through a sieve (20 mesh). Distilled water (50 mL) was added and the mixture was allowed to stand overnight at room temperature. Next, 5 mL of filtrate was obtained by filtration, and this filtrate was analysed to check for amino acids and proteins. The remaining residue and leaching solution were heated at 60 °C for 10 min. After heating, the mixture was filtered immediately. This filtrate was used to check for carbohydrates, organic acids, saponins, glycosides, phenols, tannins and cyanogenic glycosides.

Methanol extraction solutions

The powder (5 g) of LBAP or LBR was weighed and passed through a sieve (20 mesh). Ethyl ether (50 mL) was added and the mixture was heated under reflux for 10 min. The filter residue was transferred back into the bottle after filtration. Next, 35 mL of methanol was added and the mixture was heated under reflux for 10 min. After heating, the mixture was filtered immediately. This filtrate was used to check for flavonoids, anthraquinones, cardiac glycosides, coumarins, lactones, volatile oils, terpenoids, steroids, lipids and alkaloids.

Petroleum ether extraction solutions

The powder (3 g) of LBAP or LBR was weighed and passed through a sieve (20 mesh). Petroleum ether (15 mL) was added and the mixture was allowed to stand at room temperature for 4 h. Next, 5 mL of filtrate was obtained by filtration, and this filtrate was analysed to check for volatile oils, lipids, steroids and triterpenoids.

UHPLC-MS

Methanol extract of LBR was analyzed using UHPLC (Agilent 1290 system) with Q-TOF-MS (Agilent 6545 system). A ZORBAX SB-C_18_ column (150 × 3.0 mm, 1.8 µm; Agilent) was used. The column temperature was set to 40 °C. The mobile phase was a mixture of 0.1% formic acid in water (solvent A) and a mixture of 0.1% formic acid in acetonitrile (solvent B) at a flow rate of 0.4 mL/min. Linear gradient elution was applied (0–1 min, 95% A; 1–30 min, 95–70% A; 30–50 min, 70–30% A; 50–56 min, 30–1% A; 56–60 min, 1% A). The extract was diluted to 1 mg/mL with methanol and filtered using a 0.22 µm membrane before use. The sample injection volume was 5 µL. The Q-TOF-MS (Agilent) was operated in positive-ion mode with scan range *m*/*z* 100–1700. Data were recorded and analyzed with Qualitative Analysis software (version B. 07.00, Agilent).

*Stability studies of methanol extract of LBR*

pH stability

The stability in acidic and basic environments was investigated using a methanol extract of LBR dissolved in deionized water with the pH adjusted to 1, 3, 5, 7, 9, or 11 using 1 M HCl or 1 M NaOH. The final concentration of methanol extract was 50 mg/mL. After incubation at room temperature for 1 h, the pH of the mixture was adjusted to 7 and the TP_he_C and the ABTS scavenging abilities were examined.

Thermal stability

To evaluate the thermal stability, methanol extract of LBR dissolved in deionized water (50 mg/mL, pH 7) was placed in test tubes with screw caps. The test tubes were placed in a boiling water bath (100 °C). Samples were removed after 0, 15, 30, 60, 120, 180, and 240 min and cooled in an ice-water bath. and the TP_he_C and the ABTS scavenging abilities were examined.

Modeling of the stability in the gastrointestinal tract

100 mL of methanol extract of LBR in distilled water (5 mg/mL) were mixed with 10 mL of PBS (pH 6.8, 10 mM) and incubated at 37 ^o^C for 2 min (oral condition). Then 0.5 mL of 1 M HCl-KCl buffer (pH 1.5) and 5 mL of pepsin solution (pH 1.5, 32 U/mL in 1 M HCl-KCl buffer) were added to samples. The mixtures incubated at 37 ^o^C for 60 min (stomach condition). Thereafter, in the mixture was added 1 mL of 1 M NaHCO_3_ together with 1 mL of mixture of bile and pancreatic juice (pH 8.2, 10 mg/mL of pancreatin, 14,600 U/mL of trypsin, 13.5 mg/mL of bile extract in 10 mM PBS) and the pH was adjusted to 6.8. The mixtures incubated at 37 ^o^C for 3 h (duodenal condition). The results were used for determination of TP_he_C and ABTS scavenging abilities of methanol extract during simulated gastrointestinal (GI) digestion were taken at 0, 0.5, 1-4 h.

Oxidative stability of the oils

Extra virgin olive oil (EVOO) and cold-pressed sunflower oil (CPSO) were placed in separate flasks. Methanol extract of LBR was added to the EVOO and CPSO flasks at concentrations of 100 and 25 μg/g. To compare with the stabilizing effect of methanol extract, EVOO and CPSO were supplemented with synthetic antioxidants TBHQ and BHT at 200 μg/g. A control group was prepared without antioxidants. The flasks were left open and placed in an oil bath at 160 °C to simulate frying. Two samples from each category were removed from the flasks every 4 h for duplicate analysis. The oxidative stability of the oils was evaluated by measurement of the free acidity (percentage of oleic acid), peroxide values (milliequivalents of O_2_/kg oil), and ultraviolet absorption at 232 and 270 nm (K_232_ and K_270_).

Oral acute toxicity study

Twenty adult Kunming mice (19–22 g) were acquired by Liaoning Changsheng Biotechnology Co., Ltd. (animal license number SCXK (Liao) 2020–0001; Liaoning, China). Housed rats had free access to food and water under a 12 h light–dark cycle. All rats were reared adaptively for 3 days before starting the experiment. We followed the relevant policies in the Guidelines for the Use of Laboratory Animals developed by Tonghua Normal University. The Institutional Animal Care and Use Committee of Tonghua Normal University approved the experimental protocol (Ethic approval code: 20220057) and the experimental protocol follows the rules of the Declaration of Helsinki. The mice were divided into two groups (n = 10) with five males and five females in each group. The mice in the healthy control group received vehicle treatment. The methanol extract of the LB roots was dissolved in water to a final volume of 10 mL/kg mouse body weight (BW) and then administered to the mice in the LB group orally in a single dose of 2000 mg/kg LB methanol extract. The mice were then continuously observed for 1 h for behavioral changes and toxicity. Intermittent observations were made for next 6 h, and a final observation was conducted at 24 h. At this stage, the survival rate was calculated and we found that no mice died. All mice were euthanized using isoflurane. On the basis of the study results, two doses (150 and 300 mg/kg) were selected for further study.

Hepatoprotective experiments

Animals

Adult male Wistar rats (170–200 g) were acquired by Liaoning Changsheng Biotechnology Co., Ltd. (animal license number SCXK (Liao) 2020–0001; Liaoning, China). Housed rats had free access to food and water under a 12 h light–dark cycle. All rats were reared adaptively for 1 week before starting the experiment. We followed the relevant policies in the Guidelines for the Use of Laboratory Animals developed by Tonghua Normal University. The Institutional Animal Care and Use Committee of Tonghua Normal University approved the experimental protocol (Ethic approval code: 20220057) and the experimental protocol follows the rules of the Declaration of Helsinki.

Experimental protocol

All the treatments were performed daily for 7 days. After the last day of treatment, rats in groups II–V received D-galactosamine (700 mg/kg BW) by intraperitoneal injection. The rats were then fasted for 24 h with access to water. After all the above processes were completed, we found that no rats died after D-galactosamine injection. Next, all rats were anesthetized by intraperitoneal injection of pentobarbital sodium (50 mg/kg BW). Abdominal aortic blood was collected, and the hepatic tissue was rapidly excised. After leaving the blood samples at room temperature for 0.5 h, they were centrifuged for 0.25 h at 3000 rpm and 4 °C . The serum was stored at －80 °C. The tissue was washed with normal saline, dried using filter paper, and weighed. A 10% homogenate of the hepatic tissue was prepared using normal saline, centrifuged for 10 min at 10,000 rpm and 4 °C, and the supernatant was stored at －80 °C.

Histopathological examination

The hepatic tissues of three rats were selected from each group, fixed with 4% paraformaldehyde, dehydrated and washed, and embedded in paraffin. After cutting into 4-µm thick sections, hematoxylin and eosin were used for staining. Observations were made using a light microscope. A digital camera was used to record histopathological changes.

Biochemical analyses

Before anesthesia, each rat was weighed. The hepatic tissue weight and BW of each rat were used to calculate the viscera index (VI) as follows: VI = viscera weight (g)/BW (g) × 100%.

For assessment of biochemical parameters related to liver function, serum samples were analyzed for alanine aminotransferase, aspartate aminotransferase, albumin, γ-glutamyl transpeptidase, and total bilirubin. Hepatic samples were analyzed for glutathione and malondialdehyde. All samples were analyzed using commercial kits according to the manufacturer’s guidelines.

**Table S1.** Compounds identified in methanol extract of *Laportea bulbifera* roots*.*

| **Peak**  **No.** | **RT**  **(min)** | **Identification** | **Molecular**  **formula** | **Selective ion** | **Full Scan MS (m/z)** | | **Error**  **(ppm)** | **MS/MS fragments**  **(m/z)** |
| --- | --- | --- | --- | --- | --- | --- | --- | --- |
|  |  |  |  |  | **Theory** | **Measured** |  |  |
| 1 | 1.42 | N2–Fructopyranosylarginine | C_12_H_24_N_4_O_7_ | [M+H]^+^ | 337.1723 | 337.1730 | －2.1 | 236.0806, 250.0931 |
| 2 | 1.53 | Choline | C_5_H_14_NO^+^ | [M]^+^ | 104.1070 | 104.1070 | 0.0 | 104.1075 |
| 3 | 1.59 | L–Proline | C_5_H_9_NO_2_ | [M+H]^+^ | 116.0712 | 116.0707 | 4.3 | 138.0551, 133.0513 |
| 4 | 2.41 | [L–Tyrosine](javascript:treeMenu(15)) | C_9_H_11_NO_3_ | [M+H]^+^ | 182.0817 | 182.0811 | 3.3 | 165.0549, 136.0757 |
| 5 | 4.52 | L–Phenylalanine | C_9_H_11_NO_2_ | [M+H]^+^ | 166.0868 | 166.0866 | 1.2 | 120.0813 |
| 6 | 7.33 | Quinolin–2(1H)–one | C_9_H_7_NO | [M+H]^+^ | 146.0606 | 146.0605 | 0.7 | 118.0650, 130.0654 |
| 7 | 7.54 | Unknown |  |  |  | 188.0705 |  | 132.0811, 130.0657, 118.0651 |
| 8 | 7.89 | 5–Hydroxymethyl–2–furancarboxaldehyde | C_6_H_6_O_3_ | [M+H]^+^ | 127.0395 | 127.0389 | 4.7 | 109.0280 |
| 9 | 8.82 | Gallocatechin/Epigallocatechin | C_15_H_14_O_7_ | [M+H]^+^ | 307.0818 | 307.0821 | －1.0 | 289.0709, 169.0495, 139.0391 |
| 10 | 9.97 | *p*–Hydroxybenzoic acid/Salicylic acid | C_7_H_6_O_3_ | [M+H]^+^ | 139.0395 | 139.0393 | 1.4 | 121.0652 |
| 11 | 12.97 | Catechin/Epicatechin | C_15_H_14_O_6_ | [M+H]^+^ | 291.0869 | 291.0870 | －0.3 | 273.0752, 109.9437 |
| 12 | 13.84 | [Unknown](javascript:treeMenu(11)) |  |  |  | 263.1390 |  | 224.1287, 151.1234, 136.0999 |
| 13 | 14.59 | [Caffeic acid cinnamyl ester](javascript:) | C_18_H_16_O_4_ | [M+H]^+^ | 297.1127 | 297.1124 | 1.0 | 136.1005 |
| 14 | 16.37 | Isorhamnetin–7–O–*α*–L–rhamnoside/Isorhamnetin–3–O–*α*–L–rhamnoside | C_22_H_22_O_11_ | [M+H]^+^ | 463.1240 | 463.1241 | 0.2 | 431.0791, 301.0714 |
| 15 | 17.17 | [Secoisolariciresinol 9–O–β–D–glucopyranoside](javascript:) | C_26_H_36_O_11_ | [M+NH_4_]^+^ | 542.2582 | 542.2594 | －2.2 | 476.3059, 283.1083, 252.1473, 239.0814 |
| 16 | 18.34 | [Creoside IV](javascript:) | C_17_H_32_O_10_ | [M+NH_4_]^+^ | 414.2339 | 414.2350 | －2.7 | 340.2611, 295.1019 |
| 17 | 19.67 | [Schizandriside](javascript:) | C_25_H_32_O_10_ | [M+Na]^+^ | 515.1893 | 515.1889 | 0.8 | 351.1434, 189.0912 |
| 18 | 23.02 | Heptyl vicianoside | C_18_H_34_O_10_ | [M+NH_4_]^+^ | 428.2496 | 428.2510 | －3.3 | 295.1042, 133.0498 |
| 19 | 26.35 | (E)–4–Coumaric acid | C_9_H_8_O_3_ | [M+H]^+^ | 165.0552 | 165.0551 | 0.6 | 119.0603, 107.0610 |
| 20 | 32.56 | Fatty acid OH–C18:5 | C_18_H_26_O_3_ | [M+NH_4_]^+^ | 308.2226 | 308.2224 | 0.6 | 246.8626, 232.1880 |
| 21 | 35.47 | [1,4–Bis(benzoyloxy)butane](javascript:) | C_18_H_18_O_4_ | [M+H]^+^ | 299.1283 | 299.1297 | －4.7 | 194.0843, 105.0449 |
| 22 | 35.94 | Fatty acid C18:1 | C_18_H_34_O_5_ | [M+Na]^+^ | 353.2304 | 353.2309 | －1.4 | 313.2383, 299.1299 |
| 23 | 38.05 | Fatty acid OH–C18:4 | C_18_H_28_O_3_ | [M+H]^+^ | 293.2117 | 293.2118 | －0.3 | 277.2179, 265.0594 |
| 24 | 39.53 | [Nonanamide](javascript:) | C_9_H_19_NO | [M+H]^+^ | 158.1545 | 158.1543 | 1.3 | 128.1433, 114.0908 |
| 25 | 39.66 | 4–(3–Hydroxy–1–butyl)–3,5,5-trimethyl–2–cyclohexenone | C_13_H_22_O_2_ | [M+H]^+^ | 211.1698 | 211.1696 | 0.9 | 193.1589, 165.0556 |
| 26 | 40.87 | Linolenic acid | C_18_H_30_O_2_ | [M+H]^+^ | 279.2324 | 279.2323 | 0.4 | 224.1287, 165.0551 |
| 27 | 41.59 | Unknown |  |  |  | 226.1232 |  | 148.0754, 91.0542 |
| 28 | 41.87 | Palmitic acid | C_16_H_32_O_2_ | [M+NH_4_]^+^ | 274.2746 | 274.2746 | 0.0 | 240.1387, 211.2426, 128.1434 |
| 29 | 42.09 | [(Z)–9–Tetradecen–1–ol](javascript:) | C_14_H_28_O | [M+NH_4_]^+^ | 230.2484 | 230.2478 | 2.6 | 194.1169, 181.0764 |
| 30 | 42.30 | Amino fatty acid | C_18_H_39_NO_3_ | [M+H]^+^ | 318.3008 | 318.3008 | 0.0 | 256.2643, 239.0814, 224.1285 |
| 31 | 42.43 | Hydroxyhexadecanoic acid | C_16_H_32_O_3_ | [M+NH_4_]^+^ | 290.2695 | 290.2692 | 1.0 | 242.2475, 211.0867 |
| 32 | 43.79 | Fatty acid OH–C18:3 | C_18_H_30_O_3_ | [M+H]^+^ | 295.2273 | 295.2274 | －0.3 | 277.2166, 259.2048 |
| 33 | 45.25 | Fatty acid C18:4 | C_18_H_28_O_2_ | [M+H]^+^ | 277.2168 | 277.2167 | 0.4 | 178.1220, 165.0556 |
| 34 | 45.48 | 9-Octadecenedioic acid | C_18_H_32_O_4_ | [M+Na]^+^ | 335.2198 | 335.2205 | －2.1 | 295.2276, 211.1342 |
| 35 | 46.49 | Octadecanedioic acid | C_18_H_34_O_4_ | [M+Na]^+^ | 337.2355 | 337.2357 | －0.6 | 337.2357 |
| 36 | 47.42 | Unknown |  |  |  | 331.1816 |  | 353.1631, 302.3062 |
| 37 | 47.85 | Fatty acid OH–C20:0 | C_20_H_40_O_3_ | [M+NH_4_]^+^ | 346.3321 | 346.3326 | －1.4 | 299.1300, 258.2787 |
| 38 | 48.73 | [9-Hydroperoxy-10,12,15-octadecatrienoic acid](javascript:) | C_18_H_30_O_4_ | [M+H]^+^ | 311.2222 | 311.2224 | －0.6 | 293.2112, 279.2328 |
| 39 | 50.80 | Unknown |  |  |  | 488.3578 |  | 471.3320, 317.2098, 277.2169 |
| 40 | 51.07 | Fatty acid OH–C20:3 | C_20_H_34_O_4_ | [M+NH_4_]^+^ | 356.2801 | 356.2805 | －1.1 | 317.2094, 295.2277, 277.2167 |
| 41 | 51.27 | [9–HOTrE](javascript:treeMenu(9)) | C_18_H_30_O_3_ | [M+Na]^+^ | 317.2093 | 317.2095 | －0.6 | 317.2095 |
| 42 | 51.75 | Unknown |  |  |  | 317.2094 |  | 295.2272, 277.2170 |
| 43 | 53.61 | Methyl nonadecanoate | C_20_H_40_O_2_ | [M+NH_4_]^+^ | 330.3372 | 330.3367 | 1.5 | 224.1288, 128.1435 |

RT: Retention time.

In the UHPLC–MS results, peak 1 (*m/z* 337.1730) gave fragment ions at *m/z* 250.0931 and 236.0806, which was correlated with the loss of C_3_H_8_N_3_ and C_4_H_10_N_3_. These results indicated that peak 1 was N2–fructopyranosylarginine [45]. Peak 2 at *m/z* 104.1070 had a MS^2^ ion at *m/z* 104.1075 ([M]^+^), and was assigned as choline [46]. Peak 3 at *m/z* 116.0707 had a MS^2^ ions at *m/z* 138.0551 ([M+Na]^+^) and 133.0513 ([M+H_2_O]^+^), and was tentatively assigned as L–proline [47]. Peak 4 had a [M+H]^+^ peak at *m/z* 182.0811 that generated a main fragment ions at *m/z* 165.0549 ([M－NH_2_]^+^) and 136.0757 ([M－COOH]^+^), which was characteristic of L–tyrosine [48]. Peak 5 had a [M+H]^+^ peak at *m/z* 166.0866 that generated a main fragment ion at *m/z* 120.0813, and was related to －COOH loss. These results indicated that peak 5 was L–phenylalanine [49]. Peak 6 (*m/z* 146.0605) was identified as Quinolin–2(1H)–one with major MS^2^ ions at *m/z* 130.0654 and 118.0650 for the loss of －NH and －CO+H [50]. Peak 7 had an ion at m/z 188.0705 and its MS^2^ spectrum exhibited fragments at m/z 132.0811, 130.0657, and 118.0651. Peak 8 had a [M+H]^+^ peak at *m/z* 127.0389 that generated a main fragment ion at *m/z* 109.0280 ([M－OH]^+^), which was characteristic of 5–hydroxymethyl–2–furancarboxaldehyde[51]. Peak 9, a [M+H]^+^ ion at m/z 307.0821, was suggested to be either gallocatechin or epigallocatechin. Its MS^2^ ion at m/z 289.0709 ([M－OH]^+^), 169.0495 ([M－C_7_H_6_O_3_+H]^+^), and 139.0391 ([M－C_8_H_8_O_4_+H]^+^) corresponded to the literature [5]. Peak 10 was at m/z 139.0393 and characterized as *p*–hydroxybenzoic acid or salicylic acid, where its MS^2^ ion at m/z 121.0652 implied the loss of －OH [5, 51].

The main fragment ions of peak 11 (*m/z* 291.0870) appeared at *m/z* 273.0752 ([M－OH]^+^) and 109.9437 ([M－C_9_H_9_O_4_]^+^), and this peak as catechin or epicatechin [5]. Peak 12 had an ion at m/z 263.1390 and its MS^2^ spectrum exhibited fragments at m/z 224.1287, 151.1234, and 136.0999. Peak 13 (*m/z* 297.1124) was identified as [caffeic acid cinnamyl ester](javascript:) based on the typical fragment ion at *m/z* 136.1005 ([M－C_10_H_9_O_2_+H]^+^) [52]. Peak 14 (*m/z* 463.1241) gave fragment ions at *m/z* 431.0791, and 301.0714, which were correlated with the loss of －OCH_3_, and hexose. These results presumed that peak 14 was isorhamnetin–7–O–*α*–L–rhamnoside or isorhamnetin–3–O–*α*–L–rhamnoside [53]. Peak 15 with [M+NH_4_]^+^ at *m/z* 542.2594 was identified as [secoisolariciresinol 9–O–*β*–D-glucopyranoside](javascript:) based on the typical fragment ions at *m/z* 476.3059 ([M－OH－OCH_3_]^+^), 283.1083 ([M－hexose－2CH_2_OH－OH]^+^), 252.1473 ([M－hexose－2CH_2_OH－OH－OCH_3_]^+^), and 239.0814 ([M－hexose－C_7_H_7_O_2_]^+^) [54]. Peak 16 at *m/z* 414.2350 had a MS^2^ ions at *m/z* 340.2611 ([M－C_4_H_9_+H]^+^), and 295.1019 ([M－C_6_H_13_O]^+^), and was tentatively assigned as [creoside IV](javascript:) [55]. The main fragment ion of peak 17 (*m/z* 515.1889) appeared at *m/z* 351.1434 ([M－C_7_H_7_O_2_－H_2_O]^+^) and 189.0912 ([M－C_7_H_7_O_2_－C_6_H_11_O_5_－OH]^+^), and this peak was assigned as [schizandriside](javascript:) [56]. Peak 18 at *m/z* 428.2510 had a MS^2^ ion at *m/z* 295.1042 ([M－C_7_H_15_O]^+^) and 133.0498 ([C_5_H_9_O_4_]^+^), and was tentatively assigned as heptyl vicianoside [57]. Peak 19 (*m/z* 165.0551) had MS^2^ ions at *m/z* 119.0603 ([M－COOH]^+^) and 107.0610 ([M－COOH－CH_2_+H]^+^), and was identified as (E)–4–coumaric acid using previously reported data [58]. Peak 20 at *m/z* 308.2224 had a MS^2^ ion at *m/z* 246.8626 ([M－COOH－H]^+^) and 232.1880 ([M－COOH－CH_2_+H]^+^), and was assigned as fatty acid OH–C18:5 [50].

Peak 21 at *m/z* 299.1297 had a MS^2^ ion at *m/z* 194.0843 ([M－C_7_H_5_O+H]^+^) and 105.0449 ([C_7_H_5_O]^+^), and was tentatively assigned as [1,4–bis(benzoyloxy)butane](javascript:) in agreement with a previous report [59]. Peak 22 had a [M+Na]^+^ peak at *m/z* 353.2309 that generated a main fragment ion at *m/z* 313.2383 ([M－OH]^+^) and 299.1299 ([M－OH－CH_2_]^+^), which was characteristic of fatty acid C18:1 [50]. Peak 23 *(m/z* 293.2118) generated fragment ions at *m/z* 277.2179 and 265.0594, which was related to loss of －CH_3_ and －2CH_2_+H. Thus, peak 23 was assigned to fatty acid OH–C18:4 [50]. Peak 24 at *m/z* 158.1543 had a MS^2^ ion at 128.1433 ([M－C_2_H_5_]^+^) and 114.0908 ([M－C_3_H_7_]^+^), and was tentatively assigned as [nonanamide](javascript:)[60]. The main fragment ions of peak 25 (*m/z* 211.1696) appeared at *m/z* 193.1589 ([M－OH]^+^) and 165.0556 ([M－C_2_H_5_O]^+^) , and this peak was assigned as 4–(3–hydroxy–1–butyl) –3,5,5–trimethyl–2–cyclohexenone [61]. Peak 26 (*m/z* 279.2323) was identified as linolenic acid with major MS^2^ ions at *m/z* 224.1287 and 165.0551 were for the loss of －C_4_H_7_+H and －COOH－C_5_H_9_ [62]. Peak 27 had an ion at m/z 226.1232 and its MS^2^ spectrum exhibited fragments at m/z 148.0754 and 91.0542. Peak 28 had an ion at m/z 226.1232 and its MS^2^ spectrum exhibited fragments at m/z 148.0754, and 91.0542. Peak 28 at *m/z* 274.2746 had a MS^2^ ion at *m/z* 240.1387 ([M－OH+H), 211.2426 ([M－COOH]^+^), and 128.1434 ([C_9_H_19_+H]^+^), and was tentatively assigned as palmitic acid in agreement with a previous report [51]. Peak 29 *(m/z* 230.2478) was identified as [(Z)–9–tetradecen–1–ol](javascript:) with major MS^2^ ions at *m/z* 194.1169 and 181.0764 for the loss of －H_2_O and －CH_2_OH [63]. Peak 30 at *m/z* 318.3008 had MS^2^ ions at *m/z* 256.2643, 239.0814 and 224.1285 for the loss of －COOH－NH_2_, －COOH－NH_2_－OH, and －COOH－NH_2_－OH－CH_3_, respectively, and was identified as amino fatty acid [50].

The precursor ion [M+NH_4_]^+^ of peak 31 appeared at *m/z* 290.2692 and its main fragment ions were at *m/z* 242.2475 ([M－CH_2_OH+H]^+^) and 211.0867 ([M－COOH－OH+H]^+^), which corresponded to hydroxyhexadecanoic acid [51]. Peak 32 (*m/z* 295.2274) was identified as fatty acid OH–C18:3 based on the typical fragment ion at *m/z* 277.2166 ([M－OH]^+^) and 259.2048 ([M－OH－H_2_O]^+^) [50]. Peak 33 (*m/z* 277.2167) was identified as fatty acid C18:4 based on the typical fragment ions at *m/z* 178.1220 ([M－C_7_H_15_+H]^+^) and 165.0556 ([M－C_8_H_16_+H]^+^) [50]. Peak 34 at *m/z* 335.2205 had MS^2^ ions at *m/z* 295.2276 and 211.1342 for the loss of －OH and －C_5_H_9_O_2_, respectively, and was identified as 9-octadecenedioic acid [64]. Peak 35 with a [M+Na]^+^ ion at *m/z* 337.2357 was presumed to be octadecanedioic acid [65]. Peak 36 had an ion at m/z 331.1816 and its MS^2^ spectrum exhibited fragments at m/z 353.1631, and 302.3062. Peak 37 had a [M+NH_4_]^+^ ion at *m/z* 346.3326 and its main fragment ions were at *m/z* 299.1300 ([M－C_2_H_5_]^+^) and 258.2787 ([M－C_5_H_11_+H]^+^). These results were characteristic of fatty acid OH–C20:0. Peak 38 at *m/z* 311.2224 had a MS^2^ ions at *m/z* 293.2112 ([M－OH]^+^) and 279.2328 ([M－OH－CH_3_+H]^+^), and was identified as [9-hydroperoxy-10,12,15-octadecatrienoic acid](javascript:) [66]. Peak 39 had an ion at m/z 488.3578 and its MS^2^ spectrum exhibited fragments at m/z 471.3320, 317.2098, and 277.2169. The precursor ion [M+NH_4_]^+^ of peak 40 appeared at *m/z* 356.2805 and its main fragment ion was at *m/z* 317.2094 ([M－C_3_H_7_+Na]^+^), 295.2277 ([M－C_3_H_7_]^+^) and 277.2167 ([M－C_3_H_7_－H_2_O]^+^), which corresponded to fatty acid OH–C20:3. Peak 41 had a [M+Na]^+^ ion at *m/z* 317.2095 and its main fragment ion was at *m/z* 317.2095. This result was characteristic of 9–HOTrE [67]. Peak 42 had an ion at m/z 317.2094 and its MS^2^ spectrum exhibited fragments at m/z 295.2272, and 277.2170. The main fragment ions of peak 43 (*m/z* 330.3367) appeared at *m/z* 224.1288 ([M－OCH_3_－C_4_H_9_]^+^) and 128.1435 ([C_9_H_19_+H]^+^), and this peak was assigned as methyl nonadecanoate [68].


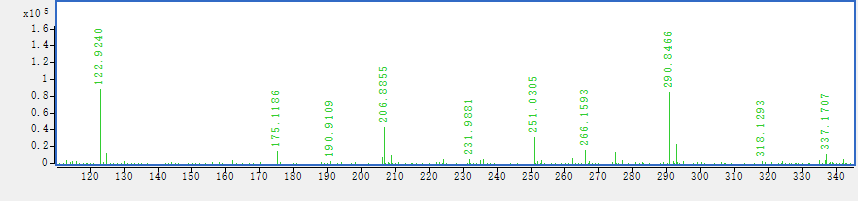


**Figure S1** MS spectrum of peak 1


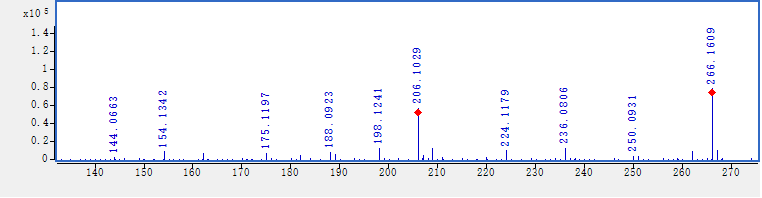


**Figure S2** MS/MS spectrum of peak 1


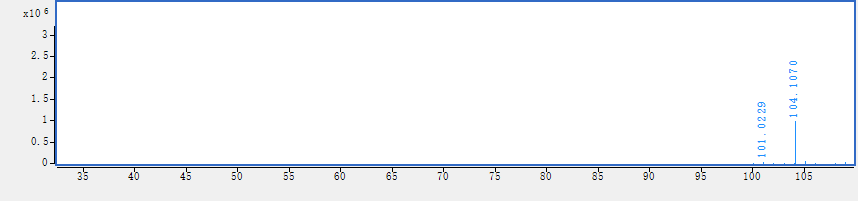


**Figure S3** MS spectrum of peak 2


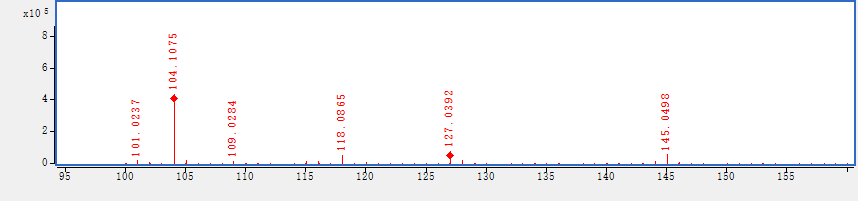


**Figure S4** MS/MS spectrum of peak 2


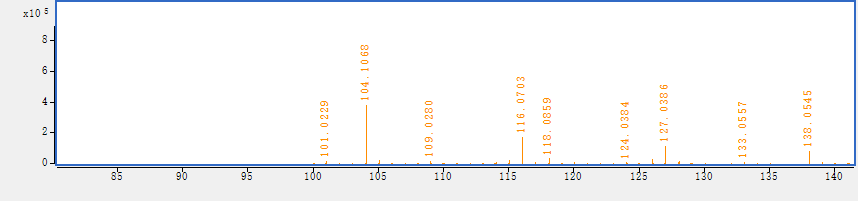


**Figure S5** MS spectrum of peak 3


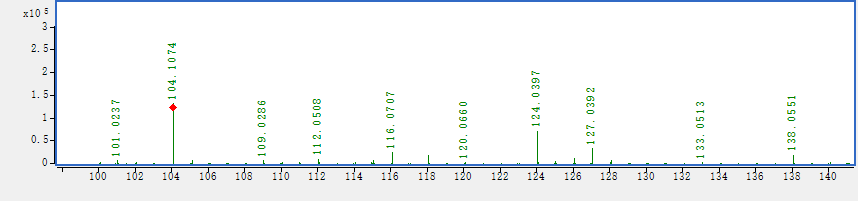


**Figure S6** MS/MS spectrum of peak 3


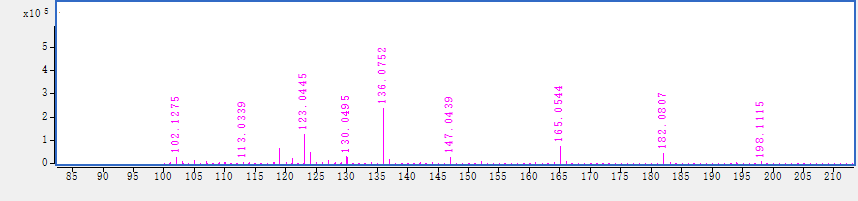


**Figure S7** MS spectrum of peak 4


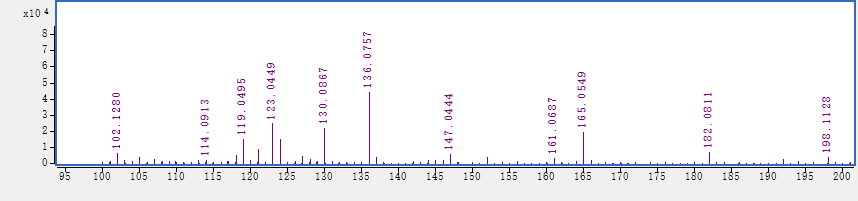


**Figure S8** MS/MS spectrum of peak 4


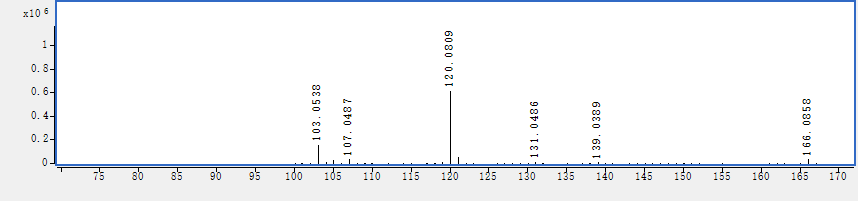


**Figure S9** MS spectrum of peak 5


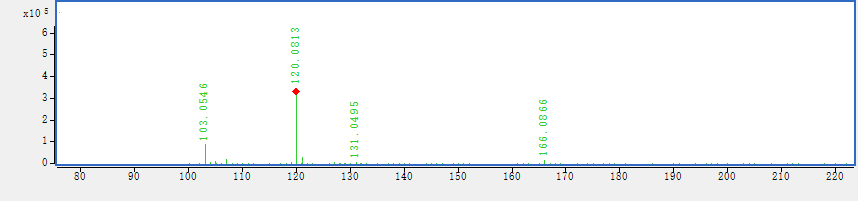


**Figure S10** MS/MS spectrum of peak 5


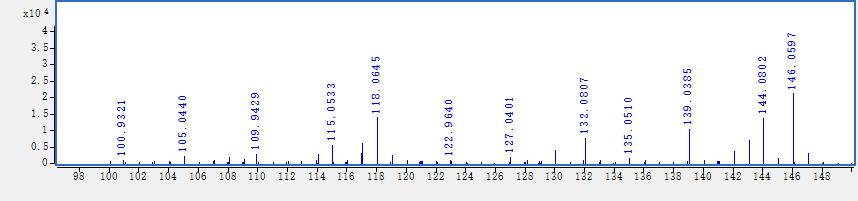


**Figure S11** MS spectrum of peak 6


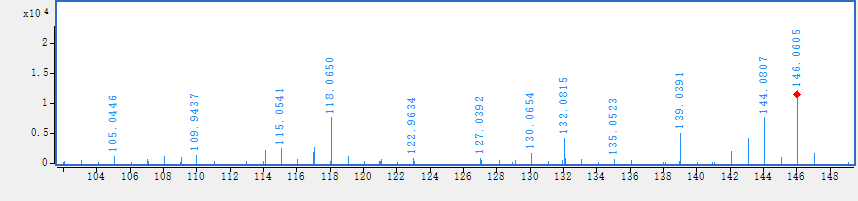


**Figure S12** MS/MS spectrum of peak 6


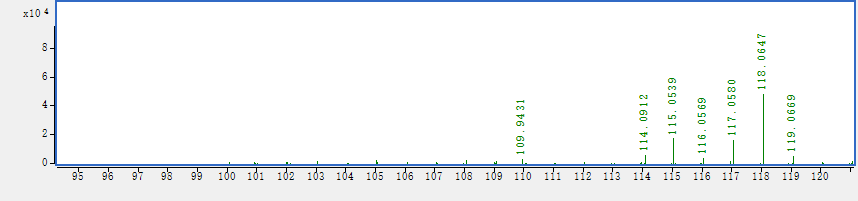


**Figure S13** MS spectrum of peak 7

**
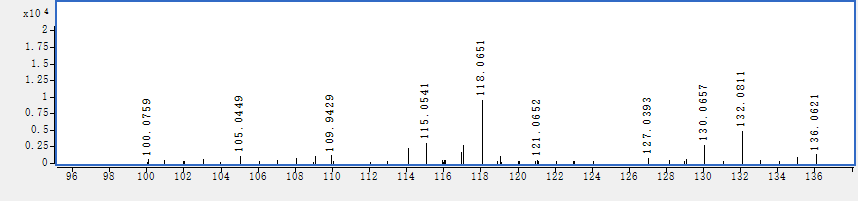
**

**Figure S14** MS/MS spectrum of peak 7


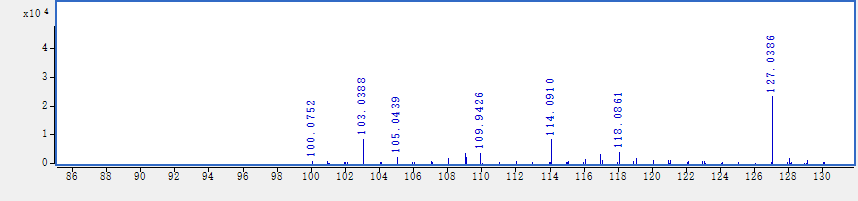


**Figure S15** MS spectrum of peak 8

**
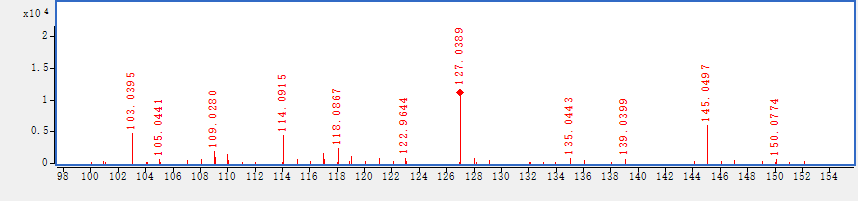
**

**Figure S16** MS/MS spectrum of peak 8


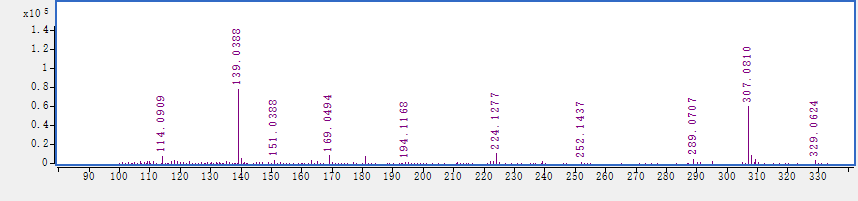


**Figure S17** MS spectrum of peak 9


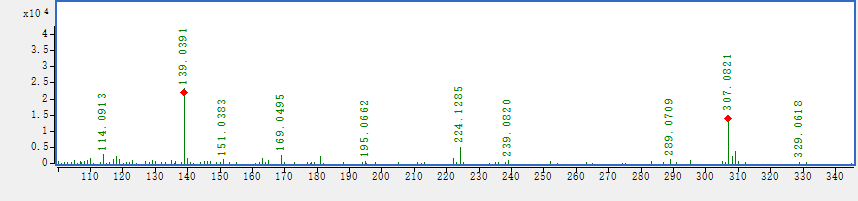


**Figure S18** MS/MS spectrum of peak 9


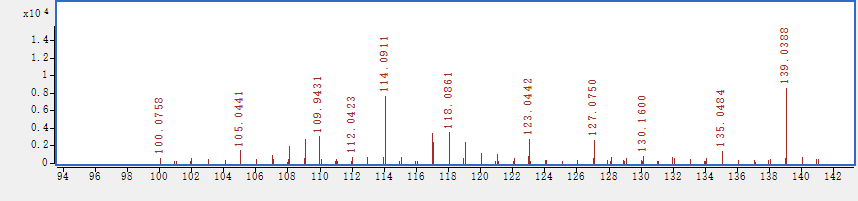


**Figure S19** MS spectrum of peak 10


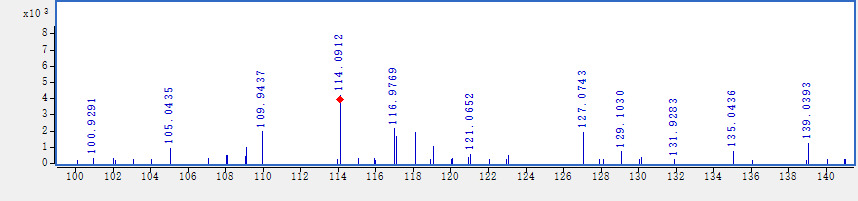


**Figure S20** MS/MS spectrum of peak 10


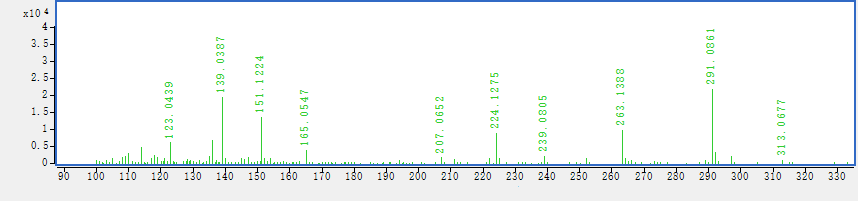


**Figure S21** MS spectrum of peak 11


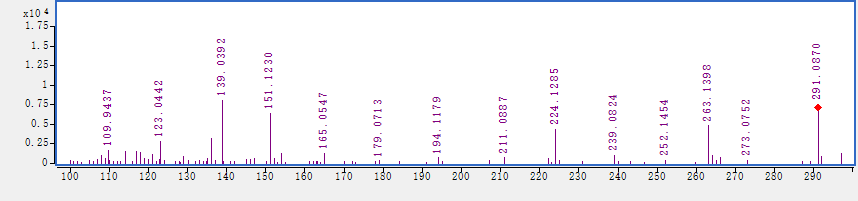


**Figure S22** MS/MS spectrum of peak 11


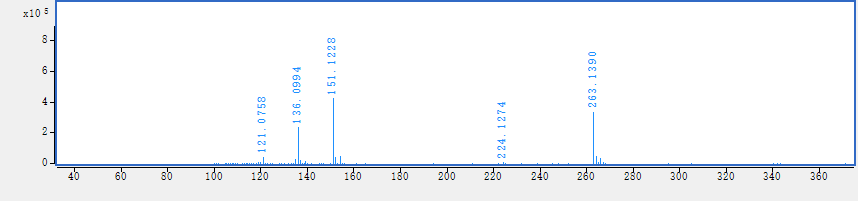


**Figure S23** MS spectrum of peak 12


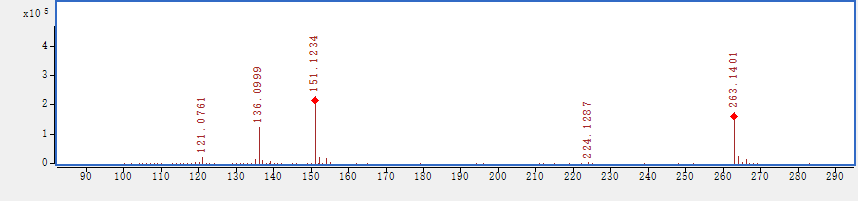


**Figure S24** MS/MS spectrum of peak 12


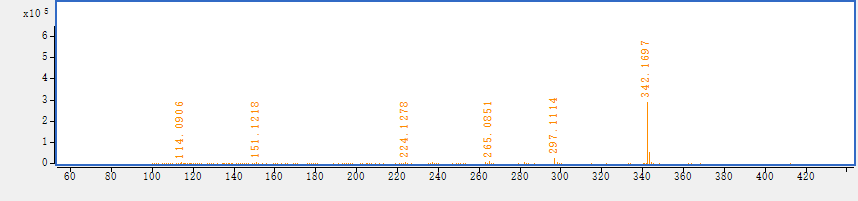


**Figure S25** MS spectrum of peak 13


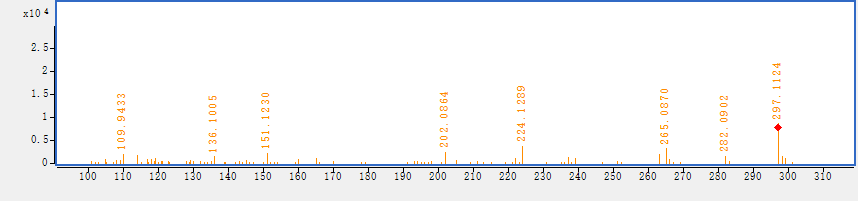


**Figure S26** MS/MS spectrum of peak 13


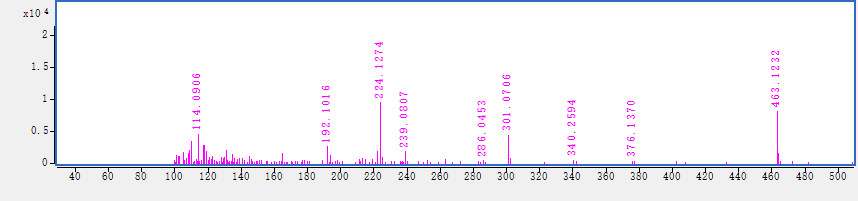


**Figure S27** MS spectrum of peak 14


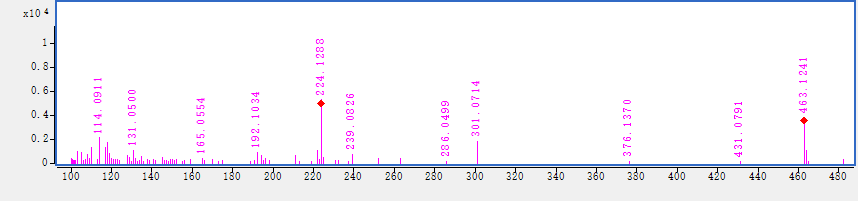


**Figure S28** MS/MS spectrum of peak 14


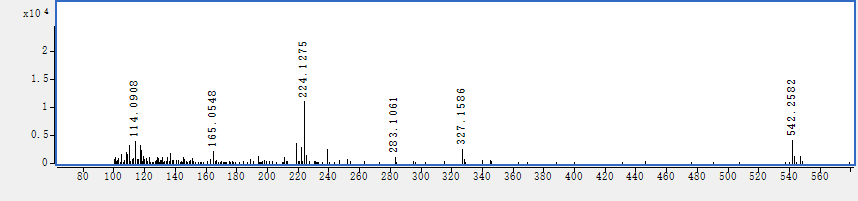


**Figure S29** MS spectrum of peak 15


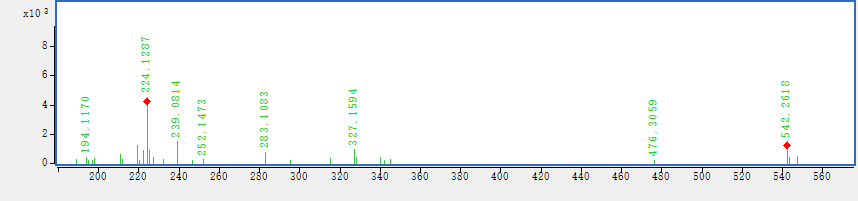


**Figure S30** MS/MS spectrum of peak 15


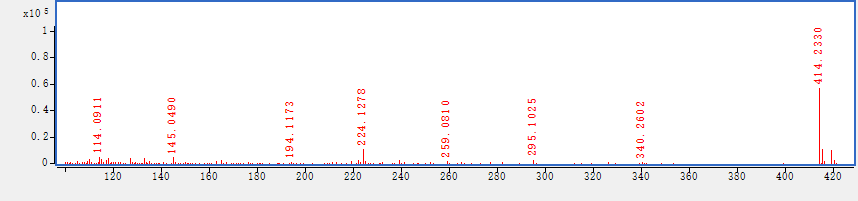


**Figure S31** MS spectrum of peak 16


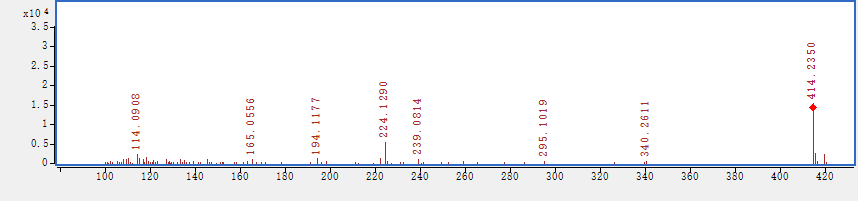


**Figure S32** MS/MS spectrum of peak 16


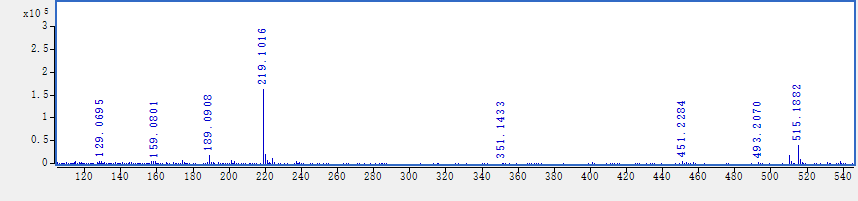


**Figure S33** MS spectrum of peak 17

**
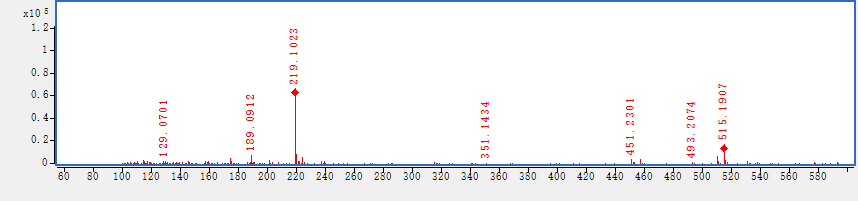
**

**Figure S34** MS/MS spectrum of peak 17


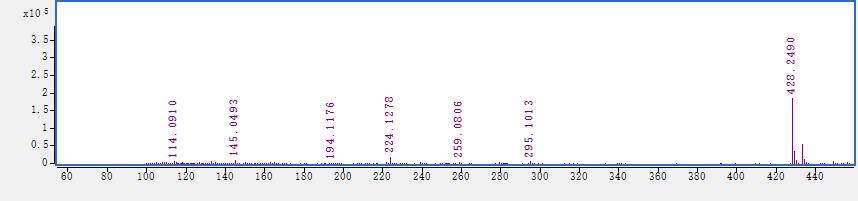


**Figure S35** MS spectrum of peak 18


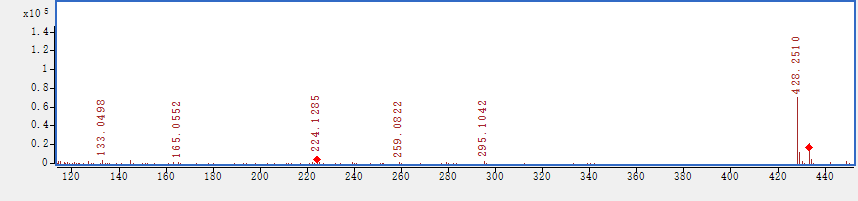


**Figure S36** MS/MS spectrum of peak 18


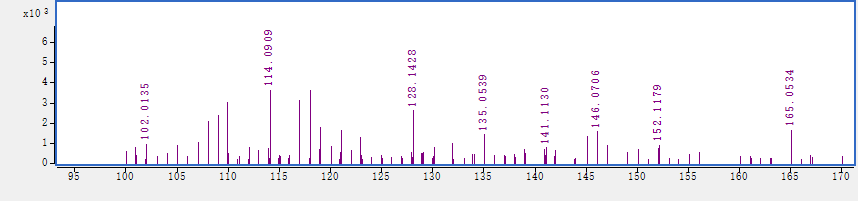


**Figure S37** MS spectrum of peak 19


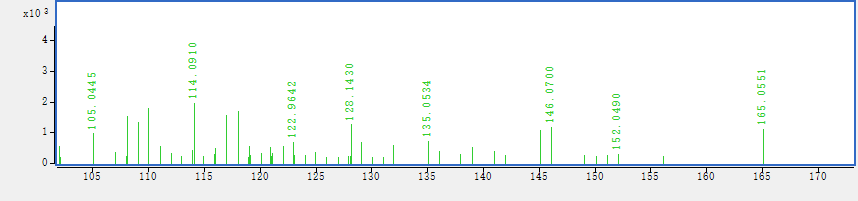


**Figure S38** MS/MS spectrum of peak 19


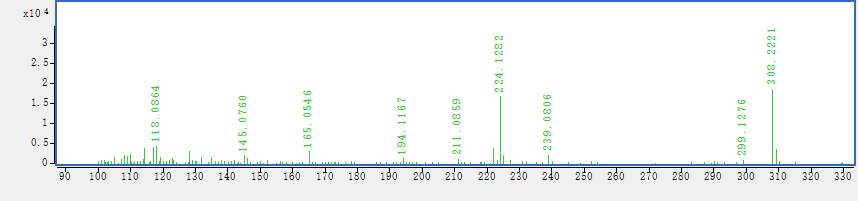


**Figure S39** MS spectrum of peak 20


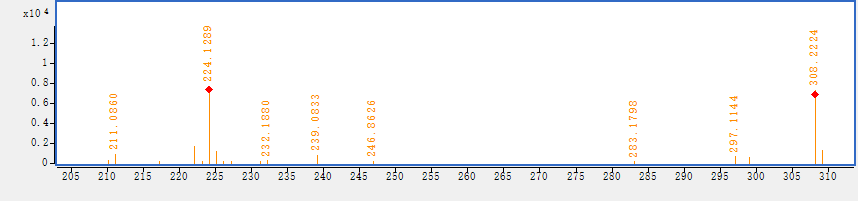


**Figure S40** MS/MS spectrum of peak 20


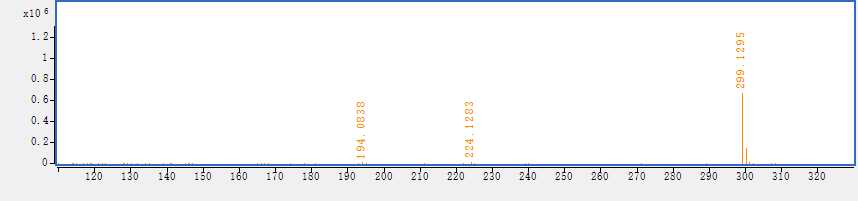


**Figure S41** MS spectrum of peak 21


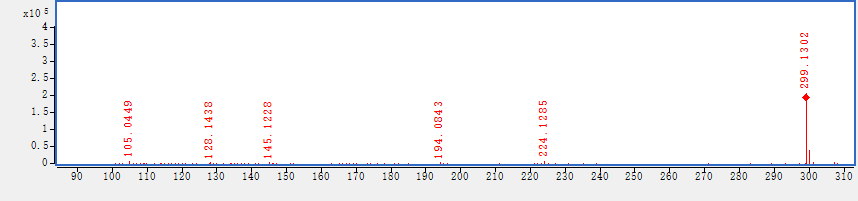


**Figure S42** MS/MS spectrum of peak 21


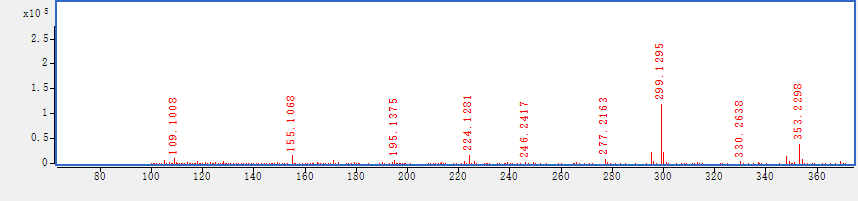


**Figure S43** MS spectrum of peak 22


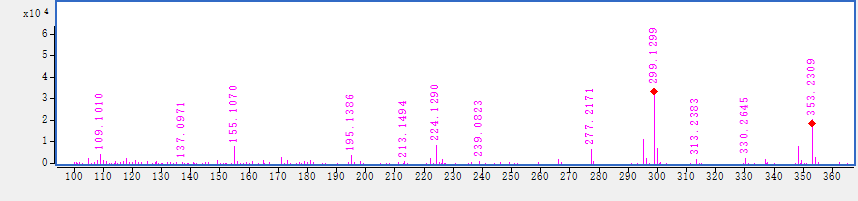


**Figure S44** MS/MS spectrum of peak 22


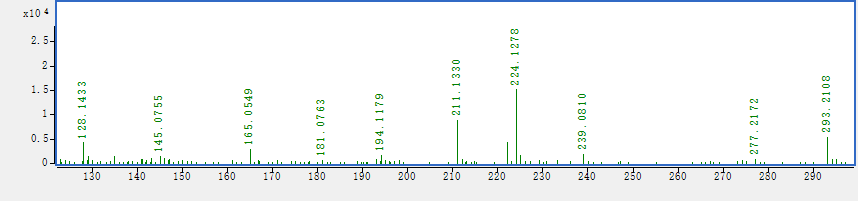


**Figure S45** MS spectrum of peak 23


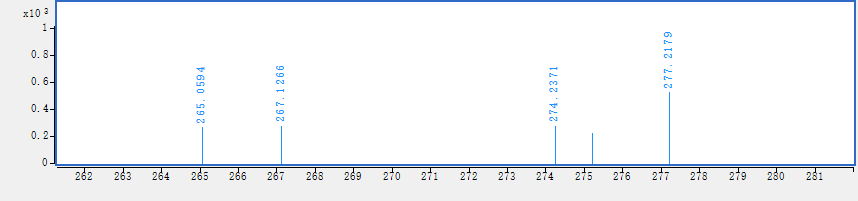


**Figure S46** MS/MS spectrum of peak 23


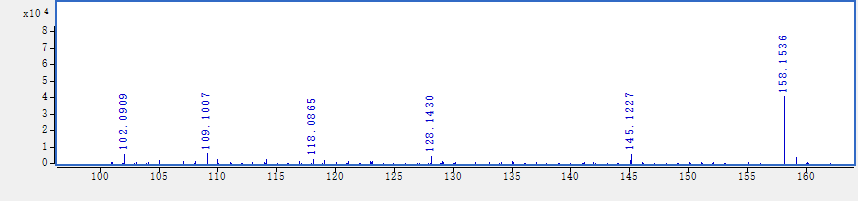


**Figure S47** MS spectrum of peak 24


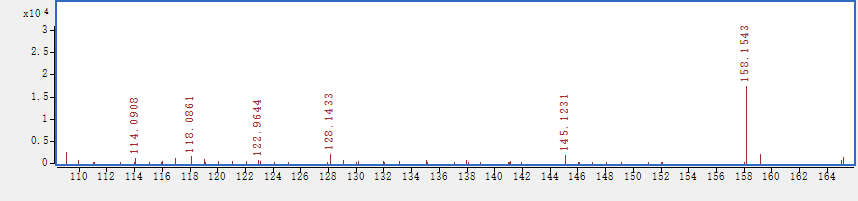


**Figure S48** MS/MS spectrum of peak 24


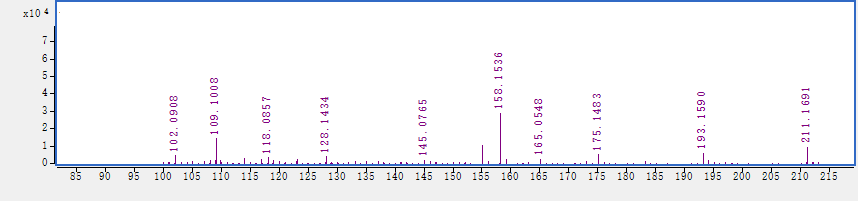


**Figure S49** MS spectrum of peak 25


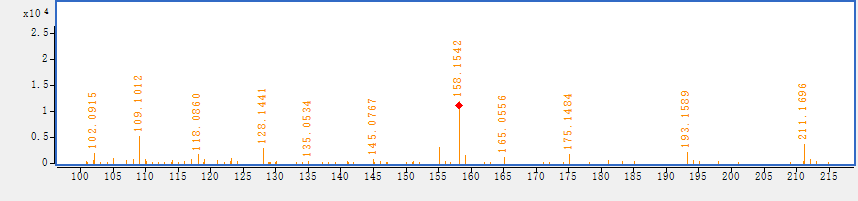


**Figure S50** MS/MS spectrum of peak 25


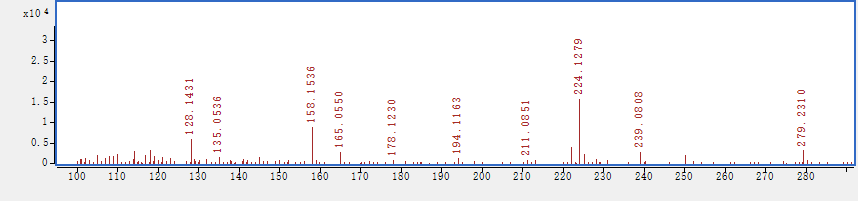


**Figure S51** MS spectrum of peak 26


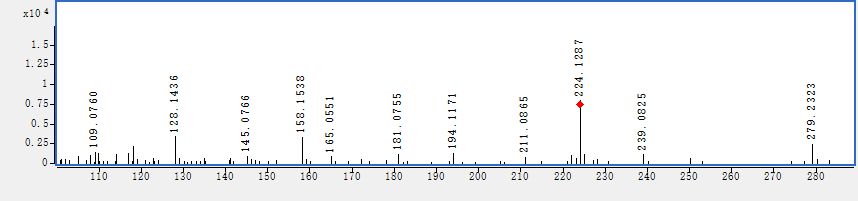


**Figure S52** MS/MS spectrum of peak 26


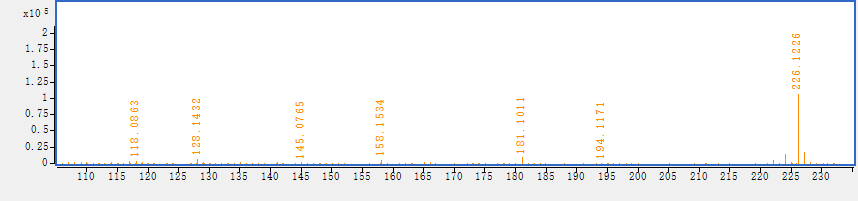


**Figure S53** MS spectrum of peak 27


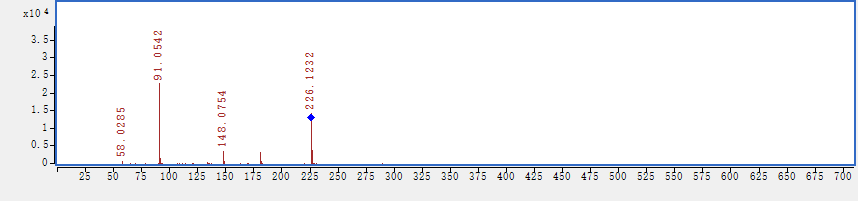


**Figure S54** MS/MS spectrum of peak 27


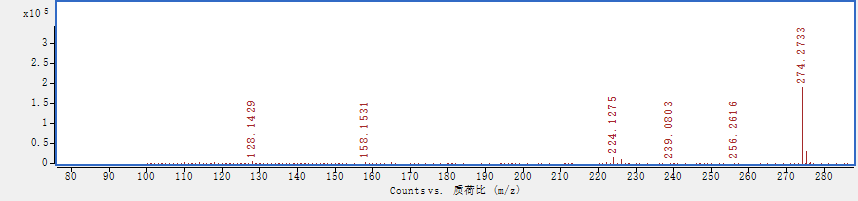


**Figure S55** MS spectrum of peak 28


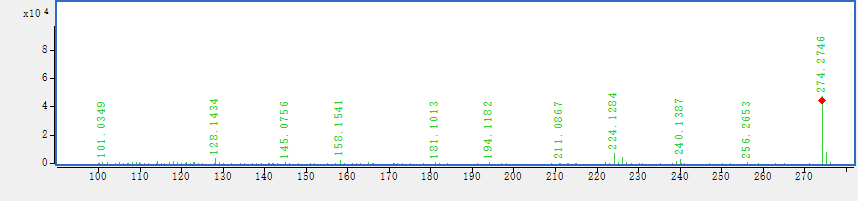


**Figure S56** MS/MS spectrum of peak 28


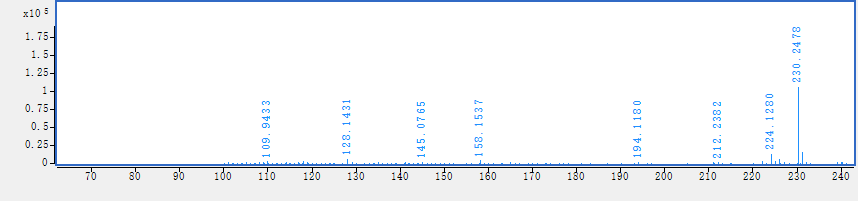


**Figure S57** MS spectrum of peak 29


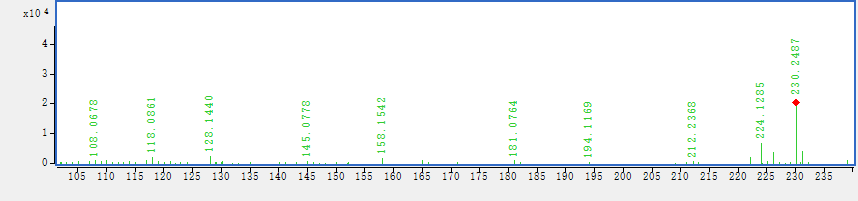


**Figure S58** MS/MS spectrum of peak 29


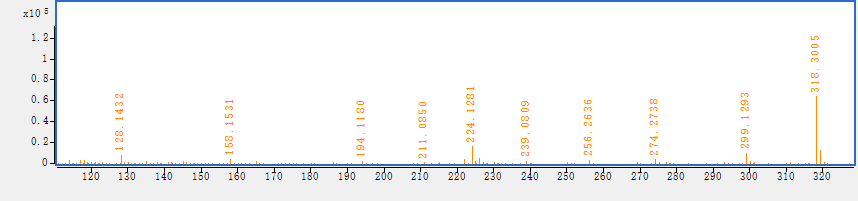


**Figure S59** MS spectrum of peak 30


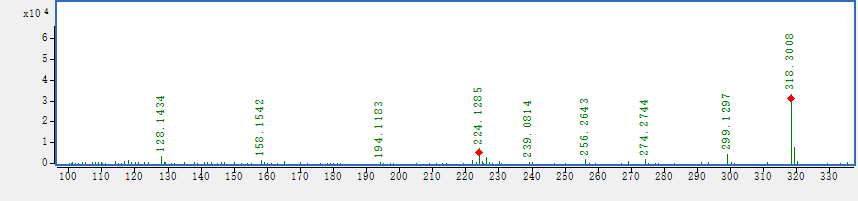


**Figure S60** MS/MS spectrum of peak 30

**
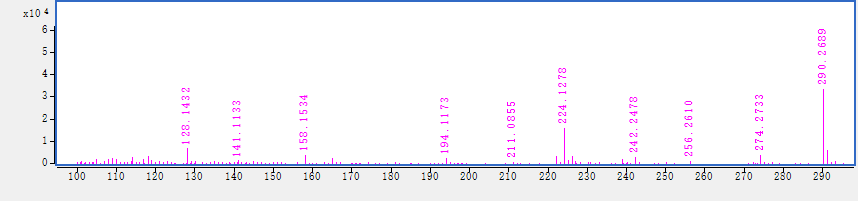
**

**Figure S61** MS spectrum of peak 31


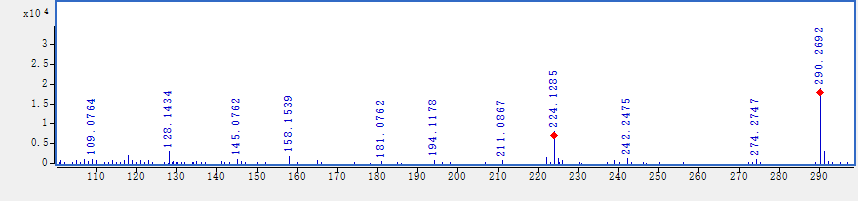


**Figure S62** MS/MS spectrum of peak 31


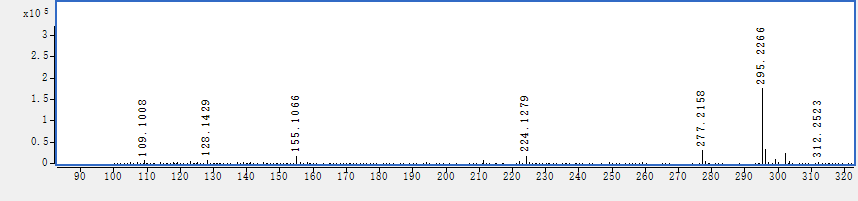


**Figure S63** MS spectrum of peak 32


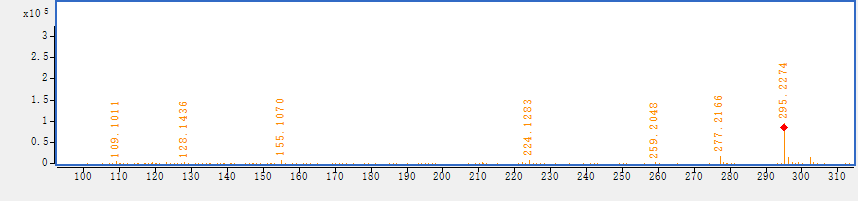


**Figure S64** MS/MS spectrum of peak 32


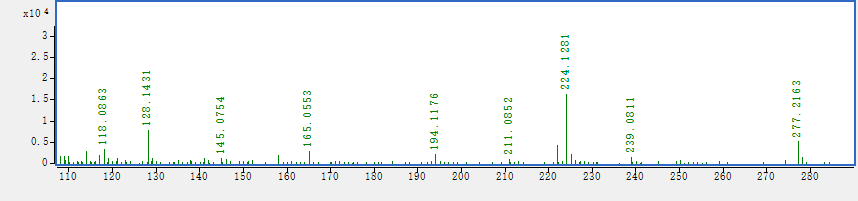


**Figure S65** MS spectrum of peak 33


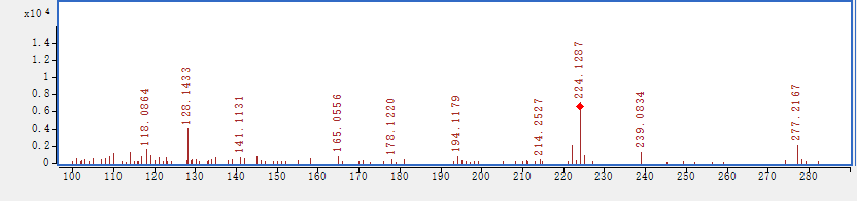


**Figure S66** MS/MS spectrum of peak 33


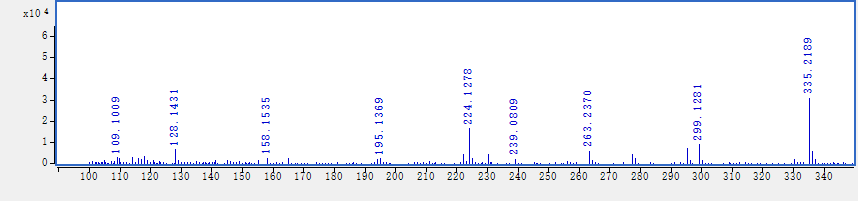


**Figure S67** MS spectrum of peak 34


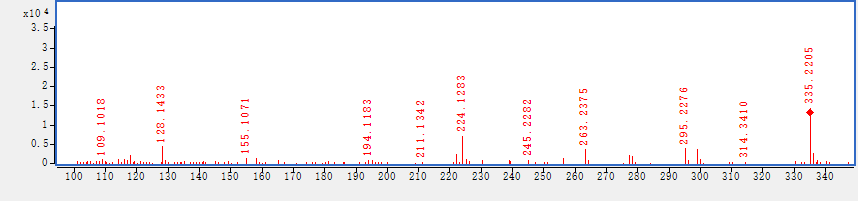


**Figure S68** MS/MS spectrum of peak 34


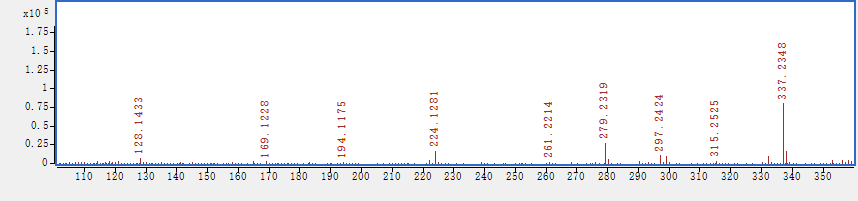


**Figure S69** MS spectrum of peak 35


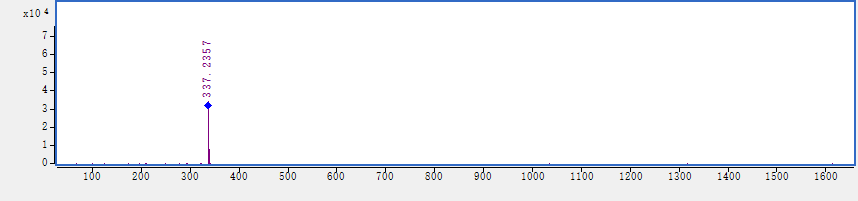


**Figure S70** MS/MS spectrum of peak 35


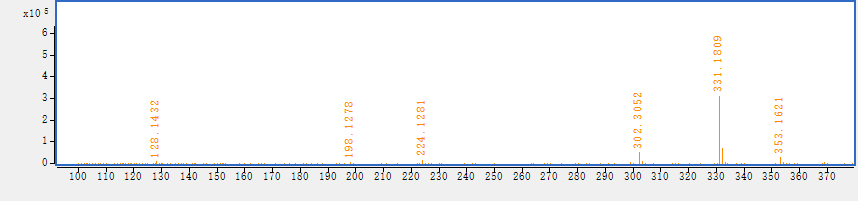


**Figure S71** MS spectrum of peak 36


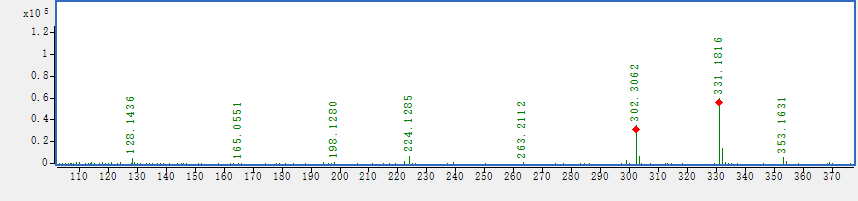


**Figure S72** MS/MS spectrum of peak 36


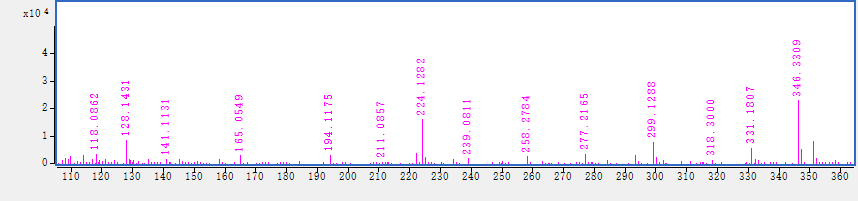


**Figure S73** MS spectrum of peak 37


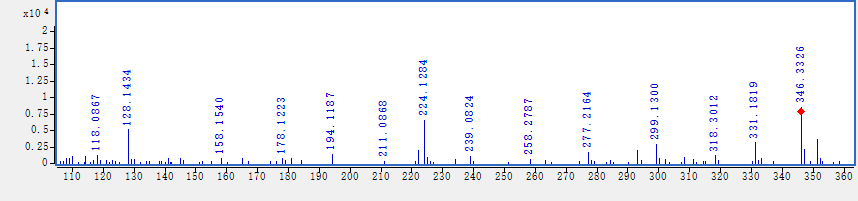


**Figure S74** MS/MS spectrum of peak 37


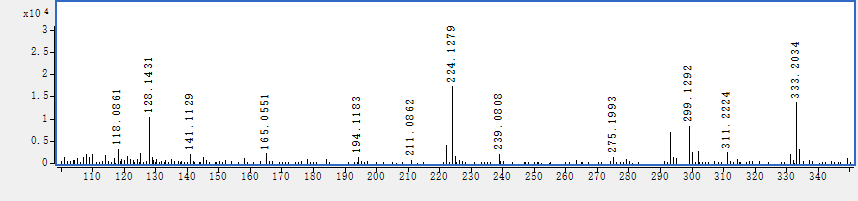


**Figure S75** MS spectrum of peak 38


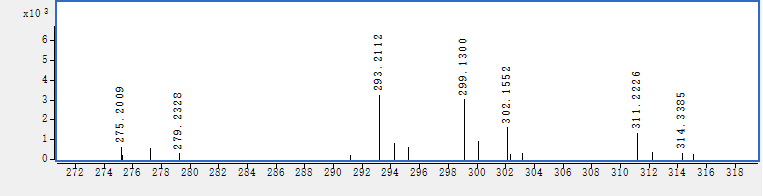


**Figure S76** MS/MS spectrum of peak 38


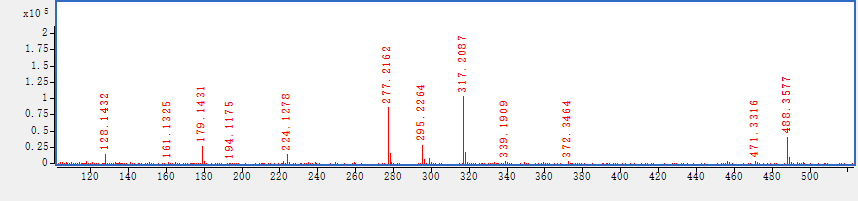


**Figure S77** MS spectrum of peak 39


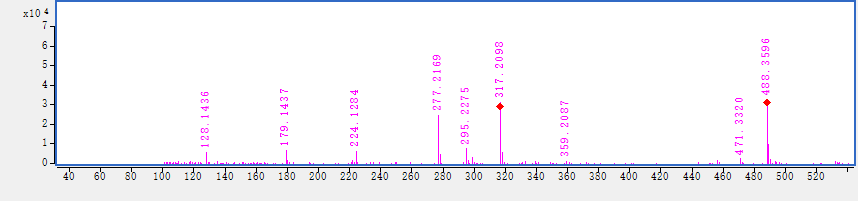


**Figure S78** MS/MS spectrum of peak 39


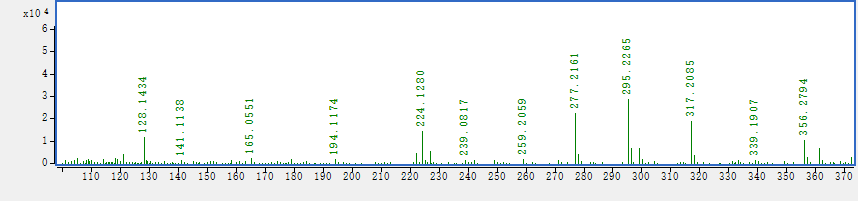


**Figure S79** MS spectrum of peak 40


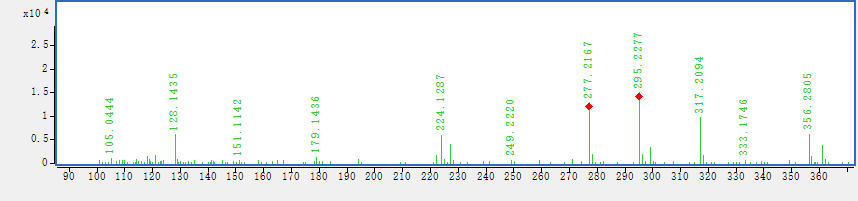


**Figure S80** MS/MS spectrum of peak 40


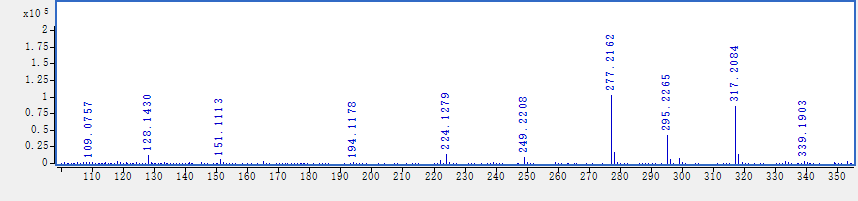


**Figure S81** MS spectrum of peak 41


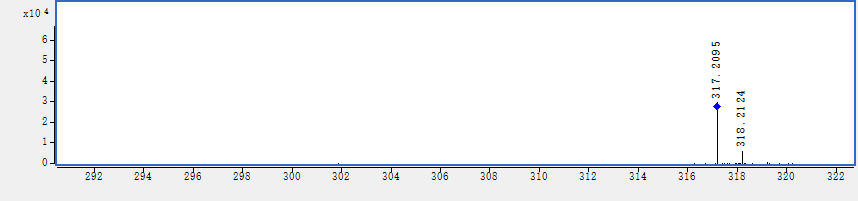


**Figure S82** MS/MS spectrum of peak 41


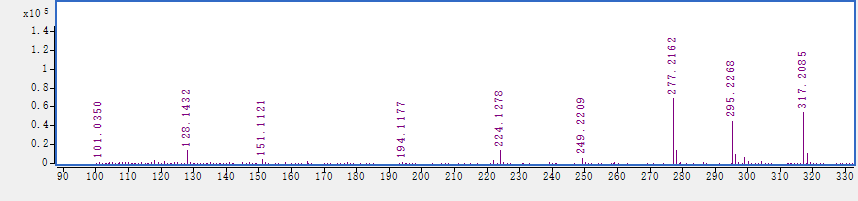


**Figure S83** MS spectrum of peak 42


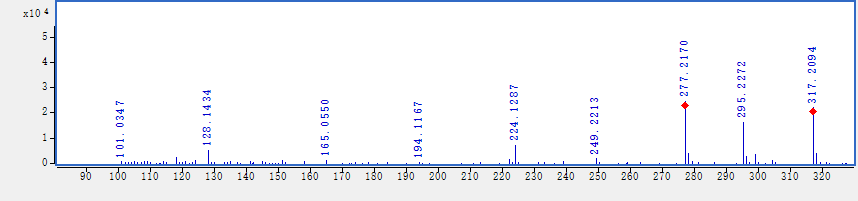


**Figure S84** MS/MS spectrum of peak 42


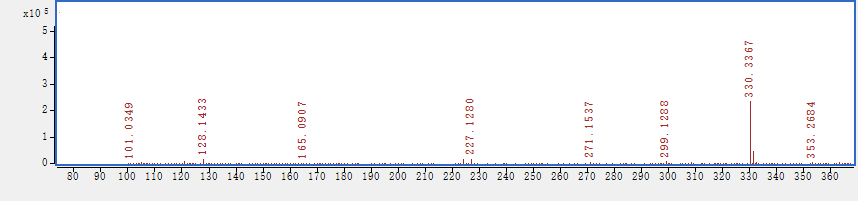


**Figure S85** MS spectrum of peak 43


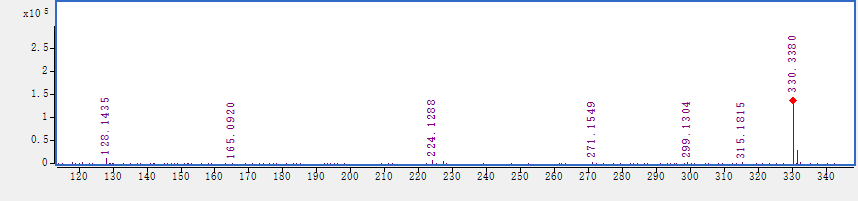


**Figure S86** MS/MS spectrum of peak 43
